# Third International Electronic Conference on Medicinal Chemistry (ECMC-3)

**DOI:** 10.3390/ph11010018

**Published:** 2018-02-09

**Authors:** Annie Mayence, Jean Jacques Vanden Eynde

**Affiliations:** 1 Haute Ecole Provinciale de Hainaut-Condorcet, 7330 Saint-Ghislain, Belgium; annie.mayence@alumni.umons.ac.be; 2 Department of Organic Chemistry (FS), University of Mons-UMons, 7000 Mons, Belgium

**Keywords:** biological and therapeutic targets, drug development, drug discovery, medicinal chemistry, nanomedicines, parasitic diseases

## Abstract

The third International Electronic Conference on Medicinal Chemistry, organized and sponsored by MDPI AG, publisher, and the journal *Pharmaceuticals*, took place in November 2017 on the SciForum website (www.sciforum.net/conference/ecmc-3). Around 300 authors from 34 different countries participated at the event, which hosted more than 70 presentations, keynotes, videos, and posters. A short description of some works presented during that scientific meeting is disclosed in this report.

## 1. Aim and Scope

As announced in our 2017 editorial [1], our journal had the opportunity to be the proud organizer of the third edition of the International Electronic Conference on Medicinal Chemistry series [2,3]. The conference took place on the internet (www.sciforum.net/conference/ecmc-3) on 1–30 November 2017. The goal of the organizers was to invite researchers involved in the field of drug discovery and drug development to present their recent works to the scientific community and to share their results with academic and industrial groups from all over the world. The event hosted more than 70 presentations, keynotes, videos, and posters. Two special round tables focused on nanomedicines, thanks to our media partner Precision Nanosystems, and on parasitic diseases, thanks to Dr. C.R. Caffrey from the University of California at San Diego (CA, USA). A brief summary of some outstanding works is presented hereafter.

## 2. Presentations, Keynotes, and Videos

### 2.1. Interaction of Zinc(II) Complexes with Relevant Nitrogen Nucleophiles under Physiological Conditions

SelimovićEnisaSoldatovicTanja*Department of Chemical-Technological Science, State University of Novi Pazar, Vuka Karadžiča bb, 36300 Novi Pazar, Serbia*Correspondence: tsoldatovic@np.ac.rs

In recent years, the design of anticancer agents has received considerable attention in the field of medicinal inorganic chemistry. Zinc(II) ion plays an important role in bioinorganic processes because of the potential formation of coordination compounds in which zinc(II) ion can readily accommodate four-, five-, or six molecules. The advantage of zinc-based anticancer drugs could be their selectivity toward specific cellular targets thanks to specific coordination ability and kinetic properties (Bertini, I., et al. *Inorg. Chem.* **1990**, *29*, 1460–1463).

The mole-ratio method was used for determining metal-ligand stoichiometry between [ZnCl_2_(en)] (where en = 1,2-diaminoethane or ethylenediamine) and imidazole at pH 7.2 in the presence of different chloride concentrations. The results indicated step-wise formation of 1:1 and 1:2 complexes in the presence of 0.010 M NaCl and 1:1 complexes in the presence of 0.001 M NaCl. Those results are correlated with additional coordination of chlorides in the first coordination sphere and with changes in coordination geometry. In the presence of 0.001 M NaCl, five-coordinate complex anion [ZnCl_3_(en)]^−^ is formed initially, and then substitution reaction with imidazole occurred. In the presence of 0.010 M NaCl the octahedral complex anion [ZnCl_4_(en)]^2−^ formed.

The kinetics of ligand substitution reactions between complex and relevant nitrogen nucleophiles such as imidazole, 1,2,3-triazole and l-histidine were investigated at pH 7.2 as a function of nucleophile concentration in the presence of 0.001 M and 0.010 M NaCl. The reactions were followed under *pseudo*-first-order conditions by UV-vis spectrophotometry. The substitution reactions included two steps of consecutive displacement of chlorido ligands and changes in coordination geometry of [ZnCl_2_(en)] complex. Results are discussed in terms of mechanisms of interactions between potential antitumor zinc-based drugs and biomolecules.

**Acknowledgments:** The authors gratefully acknowledge financial support from State University of Novi Pazar, Novi Pazar, Serbia and T. Soldatović also gratefully acknowledges financial support from Ministry of Education, Science and Technological Development, Serbia (Project No. 172011).

### 2.2. Diversity of Bioactive Endophytic Streptomyces sp. Residing in a Common Weed, Parthenium Hysterophorus (Asteraceae: Heliantheae)

TanvirRabia^1^,^2^*SajidImran^2^HasnainShahida^2^1University Diagnostic Lab (UDL), Department of Microbiology, University of Veterinary and Animal Sciences (UVAS), 54000 Lahore, Punjab, Pakistan2Department of Microbiology and Molecular Genetics, University of the Punjab, Quaid-e-Azam Campus, 54590 Lahore, Punjab, Pakistan*Correspondence: rabiatanvir@hotmail.com or rabia.tanvir@uvas.edu.pk

The sunflower family (Asteraceae) comprises of over 650 species, making it the largest plant family in Pakistan. Members of this family are extremely diverse, and include weed plants such as *Parthenium hysterophorus*.


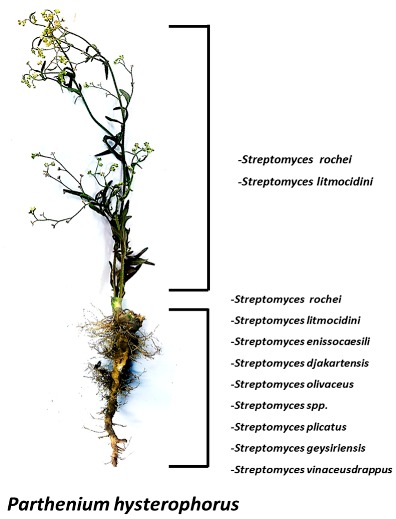


The word endophyte means ‘in the plant’, and the term includes the microbial colonizers which take up residence in the inner tissues of plants. Recently, *Streptomyces* species have been described in the plant tissues. It is noted that the possibility of re-isolation of compounds from soil actinomycetes has increased making it crucial that unexplored habitats be pursued for the search for new compounds. Considering this idea, we explored the diversity of endophytic *Streptomyces* residing in the common weed, *Parthenium hysterophorus*. A variety of *Streptomyces* sp. were identified through 16S gene sequencing that were not reported in prior studies to the best of our knowledge. The isolates were screened for their antimicrobial potential particularly against multidrug resistant (MDR) pathogens and for the diversity of their secondary metabolites through thin layer chromatography (TLC) and high-performance liquid chromatography-UV (HPLC-UV). The staining of the TLC plates by reagents such as Ehrlich’s reagent and anisaldehyde/H_2_SO_4_ revealed indoles and *N*-heterocycles, whereas the HPLC-UV chromatograms revealed peaks of diverse compounds.

### 2.3. Multifunctional Diamine AGE/ALE Inhibitors with Promising Properties for Treating Alzheimer’s Disease

LohouElodie^1^*SasakiN. André^1^BoullierAgnès^2^,^3^,^4^SonnetPascal^1^1Laboratoire de Glycochimie des Antimicrobiens et des Agroressouces (LG2A), UMR CNRS 7378, Université de Picardie Jules Verne, UFR de pharmacie, 1 rue des Louvels, F-80037 Amiens CEDEX 01, France2UFR de Médecine, Université de Picardie Jules Verne, 1 Rue des Louvels, F-80037 Amiens CEDEX 01, France3INSERM U1088, Centre Universitaire de Recherche en Santé (CURS), Avenue René Laënnec—Salouel, F-80054 Amiens CEDEX 01, France4CHU Amiens Picardie, Avenue René Laënnec—Salouel, F-80054 Amiens CEDEX 01, France*Correspondence: elodie.lohou@u-picardie.fr

Reactive carbonyl species (RCS) such as methylglyoxal (MGO) or malondialdehyde (MDA) are endogenously formed during the sugar glycoxidation and lipid peroxidation of polyunsaturated fatty acids induced by oxidative stress exacerbation. Their condensation with amino groups of tissue proteins gives Advanced Glycation Endproducts (AGE) and Advanced Lipid peroxidation Endproducts (ALE). In Alzheimer’s disease (AD), extensive AGE/ALE accumulation has been reported in extracellular amyloid *β* (A*β*) plaques and intracellular tau-associated neurofibrillary tangles. Indeed, a critical imbalance between cerebral reactive oxygen species (ROS) production and endogenous antioxidant capacities associated with biometal dyshomeostasis has been suggested to be a driving force for AD onset and progression. A*β*-oligomers induce oxidative stress whereas transition metals (Zn^2+^, Cu^2+^ and Fe^3+^) stimulate A*β* aggregation and APP (amyloid precursor protein) processing. Consequently, RCS accumulation takes part in the vicious downward redox amyloid spiral leading to neurodegeneration (Butterfield, D.A., et al. *Trends Mol. Med.* **2001**, *7*, 548–554; Tiiman, A., et al*. Neurochem. Int.* **2013**, *62*, 367–378). AGE/ALE are now considered to play an important role at the late stages of AD pathogenesis through three main mechanisms (Krautwald, M., et al. *Gerontology* **2010**, *45*, 744–751). First, glycated A*β* cross-linking promotion accelerates its deposition and its protease resistance. Secondly, AGE/ALE formation not only accelerates tau hyperphosphorylation, disturbs the neuronal membrane depolarization process and the glucose transport but also exacerbates glutamate-mediated excitotoxicity. Thirdly, AGE promote via their receptors RAGE oxidative stress and inflammation as well as cell apoptosis.

Taking into account the multifactorial pathogenesis of AD, we designed new multifunctional drugs that are simultaneously able to trap RCS (primary vicinal diamine function) as well as ROS and biometals (phenolic acid or hydroxypyridinone moiety) (Lohou, E., et al. *Eur. J. Med. Chem.* **2016**, *122*, 702–722). In the presentation, synthesis of these new promising hybrid AGE/ALE inhibitors and evaluation of their physicochemical and biological properties (carbonyl trapping capacity, antioxidant activity, Cu^2+^-chelating capacity, cytotoxicity and protective effect against in vitro MGO-induced apoptosis in the model AD cell-line PC12) are reported.


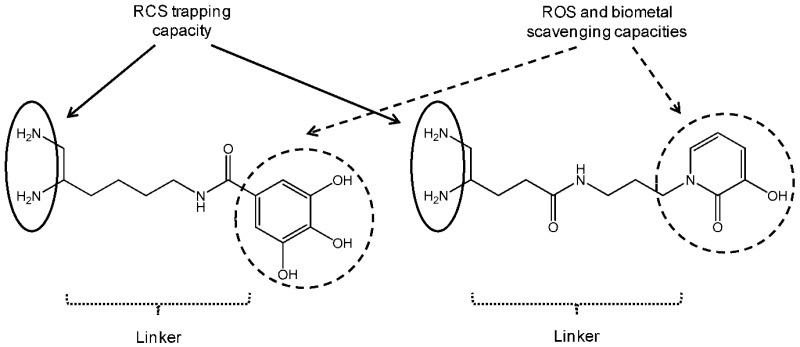


**Acknowledgments:** We thank “Société d’Accélération de Transfert de Technologies (SATT) Nord” for financial support of this study.

### 2.4. Fragments of Peptoid 1: Synthesis of N-Substituted Glycine Monomers

KohlFranziskaGütschowMichael*Pharmaceutical Institute, Pharmaceutical Chemistry I, University of Bonn, An der Immenburg 4, 53121 Bonn, Germany*Correspondence: guetschow@uni-bonn.de

Peptoids are *N*-substituted glycine oligomers with multiple biomedical applications, in particular, in nanotechnological approaches. In this context, their use is typically focused on larger oligomers, which form two-dimensional structures, but are difficult to synthesize. A short peptoid of three *N*-substituted glycine building blocks, referred to as peptoid 1, is known to inhibit the proapoptotic protein APAF1. Herein, we report on the preparation of various peptoidic building blocks of peptoid 1. The synthesis was conducted by alkylation of two different amine components, 2-(2,4-dichlorophenyl)ethylamine and 3,3-diphenylpropylamine with *tert*-butyl bromoacetate, benzyl bromoacetate, and 2-bromoacetamide, respectively. The resulting glycine derivatives have been characterized by NMR and LC/MS data. The new peptoid units will be used in biochemical studies, e.g., to evaluate protease-inhibiting properties, to perform a fragment-based approach.


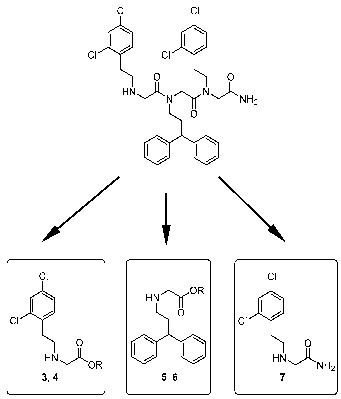


### 2.5. Interrelation between Histamine and Serotonin, Dopamine, GABA, IGF-1 in a Growth Hormone (GH) Deficient Group under rh-GH Replacement Therapy

StefanescuAna-Maria*MandaDanaPadureAdrianaDumitrescuCristina“C.I.Parhon” National Institute of Endocrinology, 34–36 Aviatorilor Blvd, 011863 Bucharest, Romania*Correspondence: stefanescuam@yahoo.com

*Aim*: To evaluate the relationship between histamine (HIST), serotonin (5-HT), dopamine (DA), gamma-amino-butyric acid (GABA) and IGF-1 in 20 GH deficient boys. *Research design and methods*: This study included 20 boys (5–14 years) with GH deficit clinically established and a 10-matched normal group with no endocrine dysfunction. All of GH deficient patients underwent GH replacement therapy. In 2017, all subjects were tested by analytical methods for blood: HIST, GABA, DA, 5-HT, IGF-1. *Results*: We divided this study group into a low HIST lot 1 (10 subjects): HIST median: 3.48 nM/L and a high HIST lot 2 (10 subjects): HIST median: 11 nM/L. Median parameters in lot 1 vs. lot 2 was: 5-HT: 212.5 vs. 370 ng/mL, DA: 30 vs. 45 pg/mL, GABA: 30 vs. 56.5 ng/mL, IGF-1: 373.5 vs*.* 200 ng/mL. Median values in normal subjects were as it follows: HIST: 5.55 nM/L; 5-HT: 235.5 ng/mL; DA: 31.5 pg/mL; GABA: 81 ng/mL. T-Test revealed a statistical significance between HIST in lot 1 vs. lot 2 (*p* < 0.001), HIST in lot 1 vs. normal group (*p* < 0.01) or HIST in lot 2 vs. normal group (*p* < 0.01). We can also underline a statistical significance between 5-HT in lot 1 vs. lot 2 (*p* < 0.05) or in lot 2 vs. normal group (*p* = 0.01). *Conclusions*: Our study underlined a HIST/5-HT positive relationship in low HIST group vs. a negative relationship HIST/5-HT in high HIST group: with small IGF-IGF-1 increments under r-GH therapy.

**Acknowledgments:** This study was approved by the Ethics Council in 2017.

### 2.6. One Year r-GH Therapy Influence on Blood Gamma-Amino-Butyric Acid, Serotonin, Dopamine and IGF-1 in 15 Growth-Hormone Deficient Children

StefanescuAna-Maria*DumitrescuCristinaPadureAdriana“C.I.Parhon” National Institute of Endocrinology, 34–36 Aviatorilor Blvd, 011863 Bucharest, Romania*Correspondence: stefanescuam@yahoo.com

*Aim*: To quantify the effect of one year r-GH therapy on blood gamma-amino-butyric acid(GABA), serotonin (5-HT), dopamine (DA) and IGF-1 in 15 growth hormone(GH) deficient children. *Research design and methods*: This retrospective study included 8 boys (7–14 years) and 7 girls (7–14 years) with clinically established GH deficit and under GH replacement therapy. In 2016 they were quantified for GABA, DA, 5-HT and IGF-1. After one year again of GH therapy they were once more tested for the same parameters using analytical methods. *Results* Median plasma parameters in 8 boys pre- vs. post-GH therapy was: GABA: 59.44 vs. 105.83 ng/mL; 5-HT: 269.66 vs. 196.55 ng/mL; DA: 46.66 vs. 91.5 pg/mL; IGF-1: 367.38 vs. 445.5 ng/mL. The same parameters were tested in 7 girls as median before and after GH therapy: GABA: 45 vs. 96 ng/mL, 5-HT: 215 vs. 200 ng/mL; DA: 40 vs. 60 pg/mL; IGF-1: 284 vs. 420 ng/mL. We established statistical significant differences in boys group before and after treatment in: plasma GABA (*p* < 0.001), serum 5-HT (*p* < 0.01), plasma DA (*p* < 0.02), serum IGF-1 (*p* = 0.02). In girls group we calculated statistical significant differences in plasma GABA before and after therapy (*p* < 0.001) and in plasma DA before and after therapy (*p* > 0.02). *Conclusions*: In fact replacement GH-therapy improved GABA/5-HT, GABA/DA, GABA/IGF-1, 5-HT/IGF-1 correlations in boys group. In girls group we estimated improved correlations between GABA/DA, 5-HT/DA, 5-HT/IGF-1. These observations could be translated in general improvement of health state in growth deficient children under GH-therapy.

**Acknowledgments:** This study was approved by the Ethics Council in 2016

### 2.7. Chiral Liquid Chromatography in Analysis of the Stereochemistry of Marine Natural Compounds: Contribution for Medicinal Chemistry

ZinWar War May^1^,^2^PrompanyaChadaporn^1^,^2^FernandesCarla^2^,^3^*CravoSara^2^,^3^PintoMadalena M.M.^2^,^3^KijjoaAnake^1^,^2^1ICBAS-Instituto de Ciências Biomédicas Abel Salazar, Universidade do Porto, Rua de Jorge Viterbo Ferreira, 228, 4050-313 Porto, Portugal2Centro Interdisciplinar de Investigação Marinha e Ambiental (CIIMAR), Universidade do Porto, Edifício do Terminal de Cruzeiros do Porto de Leixões, Av. General Norton de Matos s/n, 4050-208 Matosinhos, Portugal3Laboratório de Química Orgânica e Farmacêutica, Departamento de Ciências Químicas, Faculdade de Farmácia, Universidade do Porto, Rua Jorge Viterbo Ferreira No. 228, 4050-313 Porto, Portugal*Correspondence: cfernandes@ff.up.pt

Many naturally occurring peptides have been used in Medicinal Chemistry as pharmaceuticals or as models for drugs used in therapeutics. Marine-derived peptides are certainly an interesting source for new drugs. Taking into account the mechanisms of molecular recognition and the influence of molecular three-dimensionality in this process, it is essential to define the amino acid components of the peptide fractions isolated from marine sources.

Chiral liquid chromatography (LC) has become a very helpful and highly applicable method for preparative resolution of racemates (Sousa, M.E., et al. *J. Chromatogr. A* **2006**, *1120*, 75–81), determination of the enantiomeric purity (Silva B., et al. *Forensic Toxicol.* **2016**, 1–14), monitoring enantiomeric reactions (Fernandes, C. et al. *J. Chromatogr. A* **2012**, *1269*, 143–153), analysis of the stereochemistry of natural compounds (Prompanya, C., et al. *Mar. Drugs* **2015**, *13*, 1432–1450; Zin, W.W.M., et al., *Mar. Drugs* **2016**, *14*, 1–15), among other applications.

Herein, we describe the determination of the stereochemistry of the amino acid residues of three bioactive marine natural products (Prompanya, C., et al. *Mar. Drugs* **2015**, *13*, 1432–1450; Zin, W.W.M., et al. *Mar. Drugs* **2016**, *14*, 1–15), by chiral LC analysis of their acidic hydrolysates, using appropriate d- and l-amino acid standards. The enantioseparations of the amino acids were successfully performed on a Chirobiotic T^TM^ column under reversed-phase elution conditions. Actually, the teicoplanin selector of this column has several characteristic features that make it suitable for amino acid analysis (Berthod, A. et al. *J. Chromatogr. A* **1996**, *731*, 123–137). The elution order of all the standards amino acids enantiomers was confirmed by injecting solutions of the racemic or enantiomeric mixtures and then each enantiomer separately. The chiral LC technique was demonstrated to be decisive leading to the unambiguous elucidation of the amino acid constituents of the three bioactive marine natural products.

**Acknowledgments:** This work was partially supported through national funds from Foundation for Science and Technology (FCT) and European Regional Development Fund (ERDF) and COMPETE under the projects UID/Multi/04423/2013, PTDC/MAR-BIO/4694/2014 (POCI-01-0145-FEDER-016790), and INNOVMAR (Innovation and Sustainability in the Management and Exploitation of Marine Resources)—NORTE-01-0145-FEDER-000035, Research Line NOVELMAR. War War May Zin thanks the Lotus Unlimited Project under the ERASMUS MUNDUS ACTION 2-EU-Asia Mobility Project for a Ph.D. scholarship. Chadaporn Prompanya thanks the Faculty of Pharmaceutical Sciences, Burapha University, Thailand for her scholarship to the University of Porto. War War May Zin and Chadaporn Prompanya equally contributed to this work.

### 2.8. SAR Studies for the In Silico Prediction of HIV-1 Inhibitors

BjijImane^1^,^2^SolimanMahmoud E. S.^2^TadjerAlia^3^VilleminDidier^4^BogdanovJane^5^CherqaouiDriss^1^HdoufaneIsmail^1^*1Department of Chemistry, Faculty of Science Semlalia, BP 2390 Marrakech, Morocco2School of Health Sciences, University of KwaZulu-Natal, Westville, Durban 4000, South Africa3“ST.KLIMENT OHRIDSKI”, Faculty of Chemistry and Pharmacy, 1 James Bourchier Avenue 1164, Sofia University, 1504 Sofia, Bulgaria4Ecole Nationale Supérieure d’Ingénieurs (E.N.S.I.) I. S. M. R. A., LCMT, UMR CNRS No. 6507, 6 boulevard Maréchal Juin, 14050 Caen, France5Institute of Chemistry, Faculty of Natural Sciences and Mathematics, Ss. Cyril and Methodius University, 1000 Skopje, Macedonia*Correspondence: i.hdoufane@gmail.com

Tetrahydroimidazo[4,5,1-jk][1,4]benzodiazepines (TIBOs), as non-nucleoside analogues, constitute potent inhibitors of HIV-1 reverse transcriptase (Mandal, A.S., et al. *Eur. J. Med. Chem.* **2009**. *44*, 1509–1524). In the present study, classification structure-activity relationship (C-SAR) models are developed to distinguish between high and low anti-HIV-1 inhibitors of these compounds. Different classifiers, such as support vector machines, artificial neural networks, random forests and decision trees have been established by using ten molecular descriptors. All models were validated using several strategies: internal validation, Y-randomization, and external validation. The correct classification rate ranges from 97% to 100% and from 70% to 90% for the training and test sets, respectively. A comparison between all methods was done in order to evaluate their performances. The contribution of each descriptor was evaluated to understand the forces governing the activity of this class of compounds.


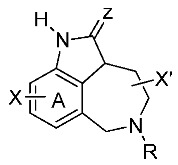


**Acknowledgments:** This work was part-supported by The “Agence Universitaire de la Francophonie” and the Scientific Research Fund of Bulgaria.

### 2.9. Synthesis, Characterization, Molecular Docking and Structure-Activity Relationships of Novel Thiazolo[3,2-a]pyrimidines as Prospective Acetylcholinesterase Inhibitors

MahgoubMohamed Y.^1^,^2^ElmaghrabyAwatef M.^2^HarbAbd-Elfttah A.^2^da SilvaJoão L. Ferreira^1^JustinoGonçalo C.^1^MarquesM. Matilde^1^*1Centro de Química Estrutural, Instituto Superior Técnico, Universidade de Lisboa, 1049-001 Lisboa, Portugal2Chemistry Department, Faculty of Science, South Valley University, Qena 83523, Egypt*Correspondence: matilde.marques@tecnico.ulisboa.pt

Acetylcholinesterase (AChE) is the enzyme that catalyzes the hydrolysis of the neurotransmitter acetylcholine (ACh) into acetic acid and choline, a crucial mechanism for regulation of neurotransmission at synapses in all nervous systems. According to the cholinergic hypothesis, depleted levels of ACh are associated with Alzheimer’s disease (Francis, P., et al. *J. Neurol. Neurosurg. Psychiatry* **1999**, *66*, 137–147).

As part of a program aimed at preparing new bioactive heterocycles with kinase and AChE inhibition properties, we designed and synthesized a series of (*Z*)-2-arylidene- and 2-amino-methylene derivatives of thiazolo[3,2-a]pyrimidine by a convenient multicomponent method. The products were fully characterized by 1D- and 2D-NMR, high resolution ESI-MS/MS and single crystal X-ray diffraction analysis, which indicated a consistent Z-configuration at the arylidene and aminomethylene double bond. Additionally, molecular docking simulations of the series of 2-arylidene/aminomethylene-thiazolo[3,2-a]pyrimidine derivatives to human AChE (PDB ID: 4m0f, chain A) were conducted to investigate the binding mode of those compounds in comparison to territrem B, used as positive control (Cheung, J., et al. *ACS Med. Chem. Lett.* **2013**, *4*, 1091–1096). The results indicate that some of the test compounds have binding energies to AChE that are comparable or better than the positive control, territrem B. Biological activity studies are underway to assess the activities of the new compounds.


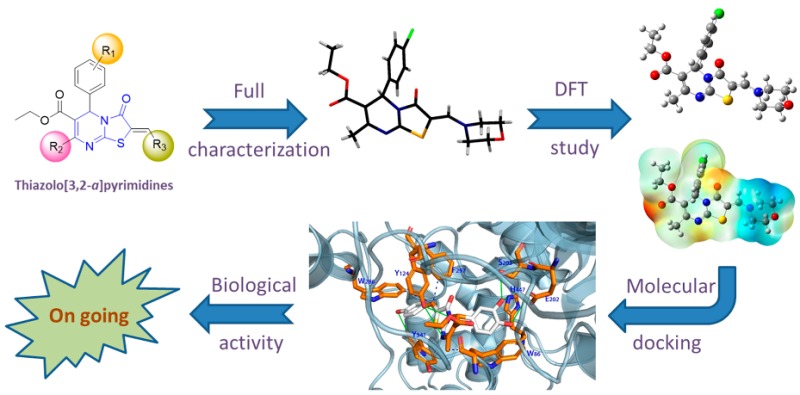


**Acknowledgments:** This work was supported in part by Fundação para a Ciência e a Tecnologia (FCT, Portugal), through grants RECI/QEQ-MED/0330/2012, RECI/QEQ-QIN/0189/2012, UID/QUI/00100/2013 and SAICTPAC/0019/2015. MYM also thanks the Egyptian Higher Education Ministry, Cultural Affairs and Missions sector for financial support. Thanks are also due to Ana Dias and Dr. Conceição Oliveira for obtaining the MS data.

### 2.10. Multivalent Engineered RNA Molecules that Interfere with Hepatitis C Virus Translation and Replication

Romero-LópezCristina^1^*LahlaliThomas^2^Berzal-HerranzBeatriz^1^Berzal-HerranzAlfredo^1^1Instituto de Parasitología y Biomedicina López-Neyra, (IPBLN-CSIC), PTS Granada, Av. Conocimiento 17, Armilla, 18016 Granada, Spain2INSERM U1052, Cancer Research Center of Lyon (CRCL), Université Claude-Bernard (UCBL), UMR_S1052, UCBL, 69008 Lyon CEDEX, France*Correspondence: cristina_romero@ipb.csic.es

The design of novel and efficient compounds fighting against the highly variable RNA viruses, such as hepatitis C virus (HCV), is a major goal. Engineering different antiviral RNAs into a single molecule yields the so-called multivalent compounds, which are promising candidates for the development of new therapeutic strategies. In this work, the previously developed chimeric inhibitor RNA HH363-10 was used as archetype for the development of improved anti-HCV inhibitors. HH363-10 consists of a hammerhead ribozyme domain, targeting the essential internal ribosome entry site (IRES) region; and an aptamer RNA molecule, directed against the highly conserved IIIf domain of the IRES. Following to the application of an in vitro selection process, new multivalent optimized chimeric anti-HCV RNA molecules derived from HH363-10 were isolated. The aptamer RNA domain was evolved to contain two binding sites: the one mapping the IIIf domain, and a newly acquired targeting site, either to the IRES domain IV (which contains the translation start codon) or the essential linker region between the IRES domains I and II. These chimeric molecules efficiently and specifically interfered with HCV IRES-dependent translation in vitro (with IC_50_ values in the low μM range). They also inhibited both viral translation and replication in cell culture. These findings highlight the feasibility of using in vitro selection strategies for obtaining improved, multivalent RNA molecules with potential clinical applications.


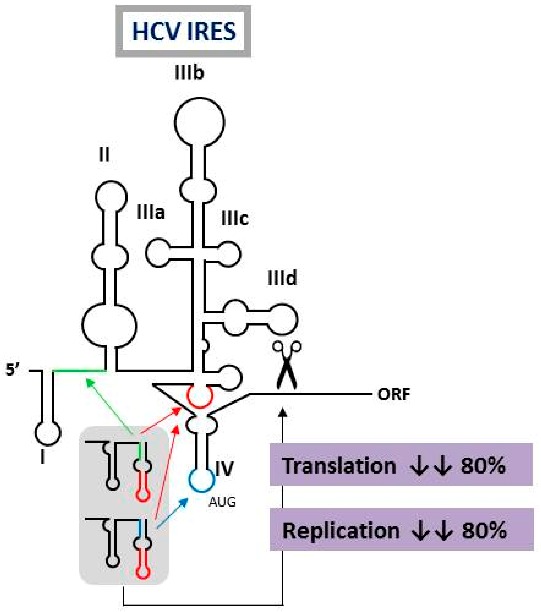


**Acknowledgments:** This work was funded by the Spanish Ministerio de Economía y Competitividad [BFU2015-64359-P]. Work at our laboratory is partially supported by FEDER funds from the EU.

### 2.11. Oligoribonucleotides-d-Mannitol Complexes Inhibit Upexpression of Genes Induced by Influenza Virus

MelnichukNataliia S.^1^*RybalkoSvetlana L.^2^TkachukZenoviy Yu.^1^1Institute of Molecular Biology and Genetics, NAS of Ukraine, 150 Zabolotnogo Str., 03680 Kyiv, Ukraine2L.V. Hromashevskyi Institute of Epidemiology and infection diseases, AMD of Ukraine, 5 Amosov str., 03038 Kyiv, Ukraine*Correspondence: natalia.melnichuk8@gmail.com

Influenza virus is an important human pathogen, which causes worldwide epidemics and pandemics. The lung is one of the most widely investigated targets for influenza virus infection and potentially at high risk of injury mediated by oxygen-derived free radicals and lipid peroxidation products (Cees, J., et al. *Free Rad. Biol. Med.* **1991**, *9*, 381–400). Influenza increases gene expression of TLR (*tlr3*, *tlr7*, *tlr8*), NF-kB (*nfκb1*, *nfκbiα*) signalings and pro-oxidation reaction (*xdh*, *nos2*) (Zou, W.L., et al. *Sci. Rep*. **2013**, *3*, 1601). The pro-oxidation gene upexpression causes overproduction of free radicals that lead to lung tissue damage (Akaike, T., et al. *Proc. Natl. Acad. Sci. USA* **1996**, *93*, 2448–2453) and the upexpression of NF-kB signaling genes induce incorrect regulation of NF-κB that regulates expression of the immune, inflammation genes and takes part in influenza viral replication (McCarty, M.F., et al. *Med. Hypotheses* **2010**, *74*, 18–20).

Oligoribonucleotides-d-mannitol complexes (ORNs-D-M) possess anti-inflammatory and anti-influenza activity in vitro and in vivo (Tkachuk, Z. U.S. Patent 20,120,232,129, 16 April 2013). In the present studies, we have found that the ORNs-D-M inhibit the upregulation of *tlr3*, *tlr7*, *tlr8, nfkbia, nfnb1 nos2*, *xdh* genes induced by influenza virus. By suppressing the upregulation of the *nos2*, *xdh* genes, the ORNs-D-M can decrease level of lipid peroxidation products at influenza virus infection in vivo. The ORNs-D-M modulate an up-expression of pro-oxidation and NF-kB signaling genes induced by influenza virus infection and are anti-influenza virus drug with effective anti-inflammation activity. By suppressing the expression of *tlr3*, *tlr7*, *tlr8*, the ORNs-D-M can impair the upregulation of *nos2*, *xdh*, *nfkbia*, *nfkb1* induced by influenza virus. The ORNs-D-M can be antagonists of the Toll-like receptors 3, 7 and 8.


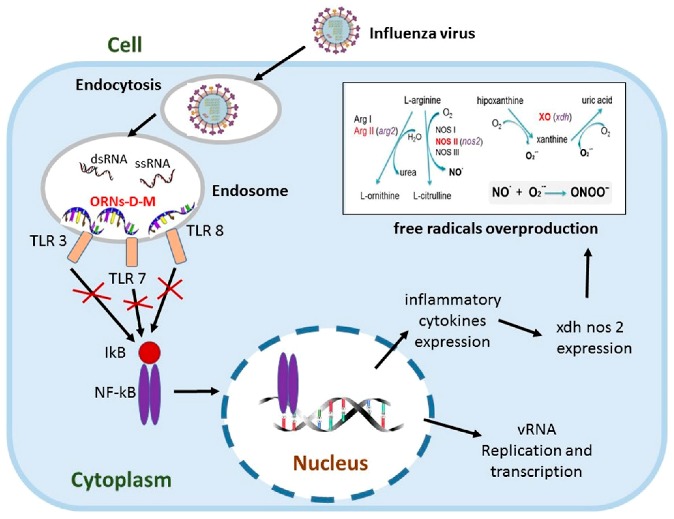


### 2.12. Analysis of Polyfluorothioacylated Amino Acids Derivatives by in silico and in vitro Methods

NaumenkoKrystyna^1^*AnnaGolovan^1^GalinaBaranova^1^YuriiShermolovych^2^SvitlanaZagorodnya^1^1Zabolotny Institute of Microbiology and Virology, National Academy of Sciences of Ukraine, Acad. Zabolotny str., 154, 03143 Kyiv, Ukraine2Institute of organic chemistry, National Academy of Sciences of Ukraine, Murmanska 5 Str., 02660 Kyiv, Ukraine*Corresponding author: krystyn.naumenko@gmail.com

Epstein-Barr virus (EBV) is the most common and persistent virus infection in humans. EBV was the first human tumour virus to be discovered (Young, L., et al. *Nat. Rev.* **2017**, *15*, 1–14). Computer-aided drug design approaches have emerged as attractive and complementary approaches to traditional high throughput screening (Li, N., et al. *Expert Opin. Drug Discov.* **2010**, *5*, 1–20). Of all commercialized pharmaceutical drugs, twenty percent contain fluorine, including important drugs in many different pharmaceutical classes. Fluorine is often added to drug molecules as even a single atom can greatly change the chemical properties of the resulting molecule in desirable ways (Swinson, J. *PharmaChem* **2005**, *25*, 26–30).


**Substances****Ра****Рі****Biological Activity****Potential Target****10S20**
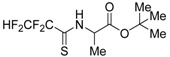
0.4850.134CYP2H substrate Dehydrogenase, mitochondrial0.3080.005Histonedeacetylase SIRT1 inhibitorHeat shock protein Hsp90-α0.2940.005Antiviral (*Picornaviruses*)Tyrosine-protein phosphatase**10S21**
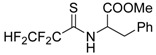
0.7150.033CYP2H substrateCell division protein kinase0.3410.057Atherosclerosis treatmentSerine/threonine protein kinase0.2140.084Antiviral (*Hepadnaviruses*)Mitogen-activated protein kinase 10**10S22**
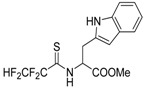
0.3970.219CYP2H substrateGag-Pol polyprotein0.2340.012Histone deacetylase SIRT1 inhibitorPurine nucleoside phosphorylaseProtein-glutamine γ-glutamyl transferase

The aim of this work was to analyze the potential biological activity and the target of action of derivatives of polyfluorothioacylated amino acids by using in silico methods and examined received results by in vitro study. For this purpose, PASS software, web-server PharmMapper, PCR, MTT assay, trypan blue and neutral red assay were used. In the present study, PASS predicted that the antiviral activity was expressed by the compound **10S20** and **10S21**. According to PASS, all studied compounds may be a substrate for cytochrome c, that might play an important role in the induction of apoptosis. It was established, that majority of the targets are enzymes, such as protein kinases and apoptotic proteins. Any predicted property must be confirmed or disproved in the biological model. Accordingly, in vitro analysis of these compounds was carried out. Our results clearly show that all derivatives are quite toxic on model Raji and B95-8 cell line. Less toxic compound **10S20**, at high concentration of 100 μg/mL exhibited a percentage of inhibition cell of 40%. Inhibition of 50 % of EBV replication was determined at minimum concentration of 1 μg/mL of compound **10S20**. SI is used to estimate the therapeutic effect of a drug and to identify drug candidates for further studies. Thus, compound **10S20** could be considered as promising new anti-EBV drug candidate for infection of EBV.

**Table:** 

**Studied Compounds**	**CC_50_^a^**	**Raji**	
		**EC_50_^b^**	**SI ^c^**
10S20	114	1	114
10S21	85	20	4
10S22	85	100	1

^a^ The 50% of studied compound for Raji and B95-8 cell in μg/mL; ^b^ Concentration of compounds (μg/mL) producing 50% inhibition of EBV reproduction; ^c^ Selectivity index (SI) = CC_50_/EC_50_

Obtained and analyzed data let to relate the compound **10S20** to a perspective anti-EBV agent, and the **10S21** and **10S22** derivatives to apoptosis-inducing compounds that can be used in further research on antitumor action.

### 2.13. Study of Antiviral Compounds in the Conditions of Mixed Infections

BiliavskaLiubov*PankivskaYuliaNaumenkoKrystynaPovnitsaOlgaZagorodnyaSvitlanaInstitute of Microbiology and Virology, National Academy of Sciences of Ukraine, Zabolotnogo str., 154, 03143 Kyiv, Ukraine*Corresponding author: bilyavskal@ukr.net

Mixed viral infection is one of the current and unexplored issues of human infectious diseases (De Palma, O., et al. *Virus Res.* **2010**, *149*, 1–9; McMahon, A., et al. *J. Biol. Chem.* **2008**, *283*, 31289–31293). The study of know drugs and discovery of new compounds using not only standard mono-infections but also with created mixed infections is a topical and a new direction in antivirus screening. Previously in our department, the models of adeno-herpetic infections in cells of different origins were created and the features of the development of viral infections in these systems were studied (Biliavska, L., et al. *Pharmaceuticals* **2015**, *9*, 14–21).

The model of simultaneous adeno-herpetic infection of MDBK cells was used for the analysis of the antiherpetic drug acyclovir (ACV) and for research of new fluorine-containing derivatives of l-phenylalanine (10S-23 and 10S-24) (Pikun, N.V. et al. *J. Fluor. Chem.* **2016**, *185*, 86–90).


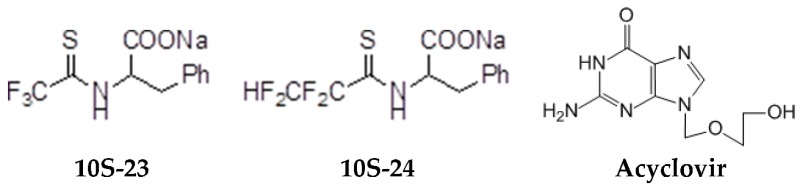


Determination of the antiviral activity accessed via real-time PCR and infectious virus yield reduction assay demonstrated the inhibitory effect of these compounds on the late stage of the HSV-1 and HAdV5 reproduction. Use of ACV under mixed infection conditions led to the 46% loss of the drug activity against HSV-1. The application of 10S-23 and 10S-24 at mixed infection induced a decrease of effectiveness of the compounds relatively herpes simplex virus by 58–73% and 16–57%, respectively. It was shown that compounds have been not effective against HAdV5 under conditions of co-infection of cells.

A comparative analysis of the cell cycle of mono- and co-infected cells allowed establishing a decrease of the acyclovir activity against herpes at the mixed infection since the increase of the apoptotic cells and the decrease of the cell number in G1-phase was detected. An abnormal action of drugs in co-infected cells indicates the need for further study of the mechanisms and targets of antiviral activity of compounds in such conditions, because using these compounds can be ineffective in medical practice (Audsley, J., et al. *Medicine* **2009**, *10*, 229–235).

**Acknowledgments:** This work was supported by a President’s of Ukraine grant for competitive projects F70 of the State Fund for Fundamental Research.

### 2.14. Study of Interactions between d-Mannitol and Polynucleotides

ShchodryiVolodymyr*TkachukZenoviyInstitute of Molecular Biology and Genetics NASU, 150 Zabolotnogo Str., 03143 Kyiv, Ukraine*Correspondence: shodryj1992@gmail.com

Complexes of oligoribonucleotides with d-mannitol are highly effective, non-toxic and with a wide range of biological effects. In particular, such complexes can increase immune reactivity and have antiviral and anti-inflammatory activity. Oligonucleotides without d-mannitol does not have such biological effects.

During the research, we have observed the decrease of fluorescence intensity of the dye water solution with polyribonucleotides. An additional decrease of intensity was observed in complexes with d-mannitol. The maximum effect was obtained for fluorescent sensor solution with polyadenylate and polycytosine. In cases of polyguanilate and polyuridine minimum changes were observed. The spectral effect may indicate about more intense interaction between polyadenylate and polycytosine with d-mannitol.

Also, we have recorded an increase in magnitude of the maximum absorption for polyadenylate and polycytosine during increasing the temperature. The value of the hyperchromic effect for polyadenylate is 10%, and 5% for polycytosine. Additional increase by the amount of hyperchromic effect was recorded for complexes with d-mannitol. Circular dichroism spectroscopy has shown spectra with a large difference in the structure of polyadenylate and polycytosine in compare with their complexes with d-mannitol. In case of polyguanilate and polyuridine and their complexes with d-mannitol, we have observed no changes in structure. Based on quantum-chemical calculations, it was established that two hydrogen bonds between d-mannitol and polynucleotides can be generated.

The obtained data allow us to assume the presence of an additional d-mannitol helix in polynucleotide and d-mannitol complexes. This assumption was also confirmed by the simulation in program package Gaussian 09. The presence of the second helix can explain a wide range of actions of this complex of polynucleotides and d-mannitol against single and two-spiral viruses.

### 2.15. Hepatoprotective Effect of Oligoribonucleotides-d-mannitol Complexes under Thioacetamide-induced Hepatotoxicity

MarchyshakTetiana^1^ShmarakovIgor^2^TkachukZenoviy^1^*1Institute of Molecular Biology and Genetics, National Academy of Sciences of Ukraine, Kyiv 03143, Ukraine2Yuriy Fedkovych Chernivtsi National University, Chernivtsi 58012, Ukraine*Correspondence: ztkachuk@bigmir.net

Therapeutic application of oligonucleotides is a leading trend in correction of metabolic disorders and related pathologies and is perceived as a unique foundation of innovative biomedicine (Sehgal, A., et al. *Hepatology* **2013**, *59*, 1354–1359). Oligoribonucleotide-d-mannitol complexes (ORNs-d-mannitol) display a vast spectrum of biological effects, including cellular metabolism stimulation with activation of endogenous synthesis of regulatory proteins, stimulation of reparation processes and ATP synthesis (Tkachuk, Z., et al. *News Farmakol. Farm.* **2009**, *3*, 14–19). Our research has been related to the determination hepatoprotective activity of ORNs-d-mannitol during acute thioacetamide-induced hepatotoxicity. This study shows that ORNs-d-mannitol decrease lesions and inflammatory infiltration of liver parenchyma under thioacetamide-induced hepatotoxicity. The ORNs-d-mannitol attenuated thioacetamide-induced free radical damage of hepatic biopolymers that is expressed in reduction of TBA-reactive products, carbonyl derivatives and in recovery of protein thiol groups, reduced glutathione. In thioacetamide toxicity it was observed that the ORNs-d-mannitol reduced the expression mRNA level of proinflammatory (interleukin 6, tumor necrosis factor α) and profibrotic (collagen type I α1, α-smooth muscle actin, transforming growth factor β1) genes, that involved in the development of hepatotoxicity. Thus, the results of this work demonstrate that the ORNs-d-mannitol have hepatoprotective effects during acute liver injury.

### 2.16. Radiolabeling Optimization and Characterization of Three 67Ga DOTA Conjugated Peptides

TassanoMarcos*CabreraMirelCabralPabloCerecettoHugoÁrea de Radiofarmacia, Facultad de Ciencias, Universidad de la República, Iguá 4225, 11400 Montevideo, Uruguay*Correspondence: mtassano@cin.edu.uy

In this work we report a preliminary study of radioactive labeling of different peptides for possible use in oncology. For this purpose the following peptides were used: KCCYSL, a probe of aberrant expression of ErbB-2, member of the epidermal growth factor family of receptors, and it has been implicated in the formation of various malignancies including ovarian cancer (Deutscher, S.L., et al. *J. Label. Comp. Radiopharm. Dec.* **2009**, *52*, 583–590); TATE, a synthetic somatostatin analog, which binds specifically to somatostatin receptors present on the cell surface of neuroendocrine tumors (Bodei, L., et al. *Eur. J. Nucl. Med.* **2011**, *38*, 2125–2135); Substance P, peptide which has an important role in modulating pain transmission trough neurokinin type 1 (NK1r) and 2 receptors (NK2r), may play a role in the pathogenesis of pancreatic tumors and malignant glial brain tumors as well (De Araújo, E.B., et al. *Cell. Mol. Biol.* **2010**, *56*, 12).


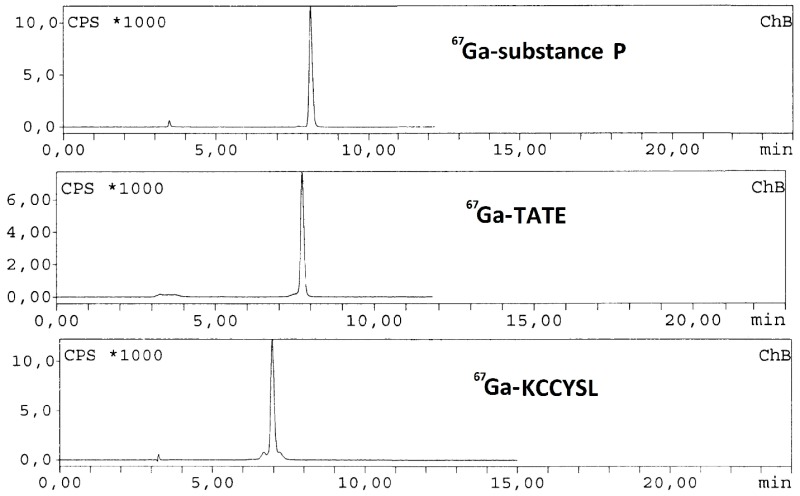


These three peptides are conjugated to DOTA 1,4,7,10-tetraazacyclododecane-1,4,7,10-tetraacetic acid) used as chelator of lanthanide ions (Min, K.M., et al. *J. Am. Chem. Soc.* **1988**, *110*, 6266–6267). Different radiolabeling methods were assayed to establish the optimum conditions for obtaining the highest yield of labeled KCCYSL, TATE and substance P. Briefly, a stock solution of the three different peptides was prepared dissolving the peptides in Milli Q water (1 mmol/L each solution). After that, 20 µg of each peptide was added to three different Eppendorf tubes containing 0.2 mL ammonium acetate buffer (pH 4.8, 0.5 mol/L) and 10 MBq of 67GaCl3 (0.02 mL/0.1 mol/L HCl) was added to a reaction solution. The reaction mixtures were kept for 30 min at 80 °C. After cooling down, the preparations were studied by HPLC (C18 reversed phase column with gradient system was used with 0.1% trifluoroacetic acid/water (Solvent A) and acetonitrile (Solvent B) as mobile phase). The three peptides were successfully labeled with high yield (>99%) under optimized conditions and remained stable for more than 48 h at room temperature.

**Acknowledgments:** The authors want to thank ANII, PEDECIBA-Quimica and PROINBIO for financial support.

### 2.17. NUC041: A Prodrug of the DNA Methyl Transferase Inhibitor (DNMTI) and Ribonucleotide Reductase Inhibitor NUC013

DaifukuRichardEpigenetics Pharma, Mercer Island, WA 98040, USA; rdaifuku@yahoo.com

NUC013 (5-aza-2′,2′-difluroro-deoxycytidine) is preclinically safer and more effective than decitabine (Daifuku, R., et al. *Pharmaceuticals* **2018**, *11*, 16). 5-Azacytdines are hydrolyzed at the cytosine’s 6-position, but in vivo, the short half-life is governed by deamination. For decitabine, attempts have been made to address these issues with continuous infusion, but use of such a regimen is limited by inconvenience and toxicity.

A hydrophobic prodrug was developed for packaging in a hydrophobic matrix to protect NUC013 from hydrolysis and deamination. In an aqueous environment, the hydrophobic moieties are readily hydrolyzed with release of NUC013. This was achieved by conjugating NUC013 with trimethylsilyl (TMS) at the 3′ and 5′ position (NUC041).

The half-life of NUC013 administered IV in mice is 20.1 min. Below, PK following administration of a dose of 3 mg of NUC041 IM in a PEG-phospholipid-depot to mice:

**Analyte****C_max_ (ng/mL)****T_max_ (h)****t½ (h)****AUC_INF_ (h·ng/mL)****MRT_INF_ (h)**NUC04142100.51.762612.6NUC013133313.458135.1

In an ongoing study, NUC041 was administered at a dose of 3 mg qwk to nude mice with NSCLC H-460 xenograft. After 3 days of treatment (*n* = 8), tumor starting volume had decreased by 4%. However, toxicity, likely from vehicle, was also observed at this dose.

### 2.18. Activity of Vitamin E Phosphate (VEP) Prodrugs of Gemcitabine in a Xenograft Model of NSCLC (NCI-H460)

DaifukuRichardEpigenetics Pharma, Mercer Island, WA 98040, USA; rdaifuku@yahoo.com

VEP nucleosides bypass two mechanisms of tumor resistance: nucleoside transport and kinase downregulation. Isoforms VE of have shown activity against solid and hematologic tumors. Gemcitabine was conjugated at the 5’ position to either δ-tocopherol-MP (NUC050) or δ-tocotrienol-MP (NUC052). NUC050 has been demonstrated to deliver gemcitabine-MP intracellularly. Its half-life IV in mice is 3.9 compared to 0.28 h for gemcitabine (Daifuku, R. *Eur. J. Cancer* **2016**, *61*, S119).

When non-small cell lung cancer tumors (NSCLC) in nude mice reached 32 to 75 mg mm^3^ (day 4) treatment was initiated with gemcitabine (120 mg/kg IP q3dx9), NUC050 or NUC052 (both 40 mg/kg qwkx4) and compared to saline control (SC).

Gemcitabine inhibited tumor growth but was not tolerated. NUC050 resulted in inhibition to tumor growth on days 11–31 (*p* < 0.05), with a nadir of −73% compared to SC. Median survival was 25.5 days (SC) vs. 33 days (NUC050) ((hazard ratio) HR = 0.24, *p* = 0.017). NUC052 had the dose increased to 50 mg/kg after 2 doses. NUC052 resulted in inhibition to tumor growth on days 14–27 (*p* < 0.05), with a nadir of −45%, and median survival was 34 days (HR = 0.27, *p* = 0.033). NUC050 and NUC052 have been shown to be safe and effective in a NSCLC xenograft.

### 2.19. Old Pharmaceuticals with New Applications: The Case Studies of Lucanthone and Mitoxantrone

HrynchakIvanna^1^Reis-MendesAna^2^PalmeiraAndreia^1^BastosMaria Lurdes^2^PintoMadalena^1^CostaVera M^2^SousaEmília^1^*1Laboratório de Química Orgânica e Farmacêutica, Departamento de Ciências Químicas, Faculdade de Farmácia. Universidade do Porto, Portugal & Centro Interdisciplinar de Investigação Marinha e Ambiental (CIIMAR), Universidade do Porto, 4099-002 Porto, Portugal2UCIBIO, REQUIMTE (Rede de Química e Tecnologia), Laboratório de Toxicologia, Departamento de Ciências Biológicas, Faculdade de Farmácia, Universidade do Porto, 4099-002 Porto, Portugal*Correspondence: esousa@ff.up.pt

The recent scope of pharmaceutical companies’ R & D programs has been undergoing some changes, especially due to increased immunopharmacology-based treatments. A trend that has also been growing is the search for new activities that may be demonstrated by drugs already used in therapeutics. Herein, examples of antitumor small molecules lead compounds obtained in our research group that arise from two existing drugs, lucanthone (**1**) and mitoxantrone (**2**, MTX) are presented.


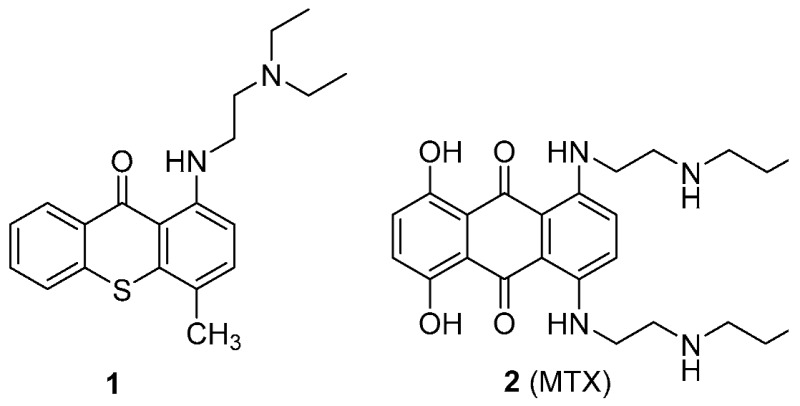


Lucanthone (**1**) was the antitumor model used to design inhibitors of P-glycoprotein with antitumor activity (Palmeira, A., et al. *Biochem. Pharmacol.* **2012**, *83*, 57–68) and activators of this transporter that could protect cells from xenobiotics were also unexpectedly discovered (Silva, R., et al. *Arch. Toxicol.* **2015**, *89*, 1783–800). Very recently, we engaged a project that intends to understand the influence of metabolites in the cardiotoxicity of an antitumor drug, MTX (**2**). Studies on the cardio-toxicity of a synthetized metabolite, naphthoquinoxaline (NAPHT) revealed that the parent drug, MTX (**2**), caused a higher disruption in the energetic pathways in a cardiac model in vitro (Reis-Mendes, A., et al. *Arch. Toxicol.* **2016**, *91*, 1871–1890). Therefore, this metabolite could be regarded as a good option for a safer anticancer therapy since it is less cardiotoxic than MTX (**2**). Following, the synthesis of the major metabolites of MTX (**2**) was performed to proceed with further toxicological studies. The examples presented herein are expected to contribute to a recent trend in drug discovery, with the involvement of old pharmaceuticals in the drug discovery process.

**Acknowledgments:** We thank FCT/MCTES and ERDF through the COMPETE–POFC programme, under the Strategic Funding UID/Multi/04423/2013, the project PTDC/MAR-BIO/4694/2014 (POCI-01-0145-FEDER-016790; 3599-PPCDT) and PTDC/DTP-FTO/1489/2014 (POCI-01-0145-FEDER-016790) in the framework of PT2020, to INNOVMAR (NORTE-01-0145-FEDER-000035, NOVELMAR), supported by NORTE 2020, under PORTUGAL 2020, through ERDF.

### 2.20. Synthesis and Tumor Cell Growth Inhibitory Effects of New Flavonosides and Xanthonosides

NevesAna R.^1^,^2^Correia-da-SilvaMarta^1^,^2^SilvaPatrícia M.A.^3^RibeiroDiana^3^SousaEmília^1^,^2^*BousbaaHassan^2^,^3^PintoMadalena^1^,^2^1Departamento de Ciências Químicas, Laboratório de Química Orgânica e Farmacêutica, Faculdade de Farmácia, Universidade do Porto, 4099-002 Porto, Portugal2Centro Interdisciplinar de Investigação Marinha e Ambiental (CIIMAR), Universidade do Porto, Porto, 4099-002 Porto, Portugal3CESPU, Instituto de Investigação e Formação Avançada em Ciências e Tecnologias da Saúde (IINFACTS), 4585-116 Gandra, Portugal*Correspondence: esousa@ff.up.pt

Natural flavonoid and xanthone glycosides display several biological activities (Leong, C.N.A., et al. *Food Chem.* **2008**, 109, 415–420, Kumar, M., et al., *Fitoterapia* **2010**, *81*, 234–242, Reutrakul, V., et al. *Planta Medica* **2007**, *73*, 683–688), with the glycoside moiety playing an important role in the mechanisms of action of these secondary metabolites. Herein, to give further insights into the inhibitory cell growth activity of these classes of compounds, the synthesis of new flavonoid and xanthone derivatives containing one or more acetoglycoside moieties was carried out to evaluate their in vitro cell growth inhibitory activity in human tumor cell lines.


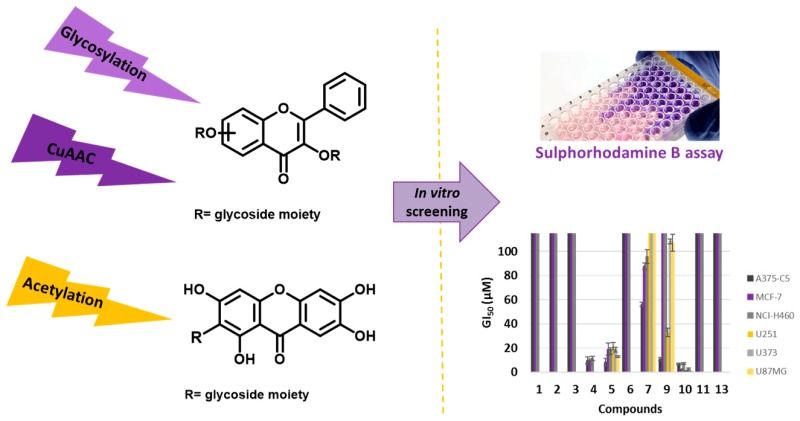


The introduction of one or two acetoglycoside moieties in the framework of a hydroxylated flavonoid was performed using three synthetic methodologies: Michael reaction, Koenigs-Knorr reaction, and through a copper catalyzed azide-alkyne cycloaddition. Acetyl groups were introduced using acetic anhydride under microwave irradiation. The in vitro cell growth inhibitory activity of seven synthesized compounds were investigated in six human tumor cell lines: A375-C5 (malignant melanoma IL-1 insensitive), MCF-7 (breast adenocarcinoma), NCI-H460 (non-small cell lung cancer), U251 (glioblastoma astrocytoma), U373 (glioblastoma astrocytoma), and U87MG (glioblastoma astrocytoma). The most active compound in all tumor cell lines was a flavonoside and showed GI_50_ values below 10 μM.

**Acknowledgments:** This work was supported through national funds provided by Foundation for Science and Technology from the Minister of Science, Technology and Higher Education (FCT/MCTES—PIDDAC) and European Regional Development Fund (ERDF) through the COMPETE—Programa Operacional Factores de Competitividade (POFC) (POCI-01-0145-FEDER-016790), PPCDT—Promover a Produção Científica e Desenvolvimento Tecnológico e a Constituição de Redes Temáticas (Project 3599), under the project PTDC/MAR-BIO/4694/2014 in the framework of the programme PT2020 and INNOVMAR—Innovation and Sustainability in the Management and Exploitation of Marine Resources, reference NORTE-01-0145-FEDER-000035, Research Line NOVELMAR in the framework of North Portugal Regional Operational Programme (NORTE 2020). Marta Correia-da-Silva thanks FCT for the postdoctoral fellowship SFRH/BPD/81878/2011 and Ana R. Neves and Patrícia M.A. Silva for the Ph.D. fellowships SFRH/BD/114856/2016 and SFRH/BD/90744/2012, respectively.

### 2.21. Procedures for the GMP-Compliant Production and Quality Control of [^18^F]PSMA-1007: A Next Generation Radiofluorinated Tracer for the Detection of Prostate Cancer

NeelsOliver C.^1^*MartinRené^2^CardinaleJens^3^SmitsRené^2^SchäferMartin^1^HoeppingAlexander^2^MüllerMarco^2^KopkaKlaus^1^1German Cancer Research Center, Im Neuenheimer Feld 280, 69120 Heidelberg, Germany2ABX advanced biochemical compounds, Heinrich-Glaeser-Strasse 10-16, 01454 Radeberg, Germany3Ludwig Boltzmann Institute Applied Diagnostics, Waehringer Guertel 18-20, 1090 Vienna, Austria*Correspondence: o.neels@dkfz.de

Radiolabeled tracers targeting the prostate-specific membrane antigen (PSMA) have become important radiopharmaceuticals for the PET-imaging of prostate cancer. In this connection, we recently developed the fluorine-18-labelled PSMA-ligand [^18^F]PSMA-1007 as the next generation radiofluorinated Glu-ureido PSMA inhibitor after [^18^F]DCFPyL and [^18^F]DCFBC. Since radiosynthesis so far has been suffering from rather poor yields, novel procedures for the automated radiosyntheses of [^18^F]PSMA-1007 have been developed. We herein report on both the two-step and the novel one-step procedures, which have been performed on different commonly-used radiosynthesisers. Using the novel one-step procedure, [^18^F]PSMA-1007 was produced in good radiochemical yields ranging from 25 to 80% and synthesis times of less than 55 min. Furthermore, upscaling to product activities up to 50 GBq per batch was successfully conducted. All batches passed quality control according to European Pharmacopoeia standards. Therefore, we were able to disclose a new, simple and, at the same time, high yielding production pathway for the next generation PSMA radioligand [^18^F]PSMA-1007. Actually, it turned out that the radiosynthesis is as easily realised as the well-known [^18^F]FDG synthesis and, thus, transferable to all currently-available radiosynthesisers. Using the new procedures, the clinical daily routine can be sustainably supported in-house even in larger hospitals by a single production batch.

### 2.22. Reactivity under Microwave Irradiation of 2-Amino-4H-Chromene-3-Carbonitrile as Tool for the Construction of Potential Bioactive Derivatives

BazureauJean-Pierre^1^,^2^*BouattourAli^3^FakhfakhMehdi^3^AbidSouhir^3^AmmarHoucine^3^PaquinLudovic^1^,^2^GuévelRémy Le^4^CorluAnne^4^RuchaudSandrine^5^BachStéphane^5^1Institut des Sciences Chimiques de Rennes, ISCR UMR 6226, groupe CORINT, Université de Rennes 1, 35042 Rennes CEDEX, France2Université de Rennes 1, S2 Wave platform, ScanMat UMS 2001 CNRS, 35042 Rennes CEDEX, France3Laboratoire de Chimie Appliquée: Hétérocycles Corps Gras & Polymères, Université de Sfax, 3000 Sfax, Tunisie4ImPACcell platform, SFR Biosit, Université de Rennes 1, 35043 Rennes CEDEX, France5Station Biologique de Roscoff, USR 3151, CNRS-UPMC, *Kissf* platform, 29682 Roscoff, France*Correspondence: jean-pierre.bazureau@univ-rennes1.fr

The interesting 4*H*-chromenes (or 4*H*-benzopyranes) and their derivatives are components of many naturally occurring products, which have also been submitted to structural modifications to increase molecular diversity, for potential medicinal properties. In this context and starting from the 2-amino-2*H*-benzopyran-3-carbonitrile platform, it was possible to easily build (in 20 min) a new class of 4-imino-3-phenyl-3,4-dihydro-1*H*-chromeno[2,3-*d*]pyrimidine-2(5*H*)-thiones in good yield (55–70%) under microwave irradiation at 120 °C in pyridine medium (Bouattour, A., et al. *Synthesis* **2017**, 49, 3768–3774). Treatment of the amino 4*H*-chromene platform with orthoester gave the corresponding methanimidate intermediate, which is converted into formamidine derivatives (63–85%) using various cyclic secondary amines or, into *N*-3 substituted 4*H*-benzopyrano[2,3-*d*]pyrimidine-4(5*H*)-imines (49–94%) under microwave irradiation (50–120 °C, 30 min) (Bouattour, A., et al. *Arkivoc* **2017**, *iv*, 291–302). The biological properties of all products were explored by in vitro cancer assays against a panel of seven tumor cell lines (Huh 7D12, Caco2, MDA-MB231, HCT116, PC3, NCI-H727, HaCat, fibroblasts which are representative of different cancers: leukemia, melanona, and cancers of liver, colon, breast, prostate, lung, and kidney) and also, in vitro serine/threonine protein kinase inhibition assays (*Hs*CDK5-p25, GSK3α/β, CLK1, *Hs*Haspin, *Hs*PIM1, *Hs*Aurora B). Some of these 2-imino- or 2-amino-2*H*-benzopyran-3-carbonitriles are active against tumor cell lines (Huh7, Caco 2, HCT 116) or protein kinases (CLK1).


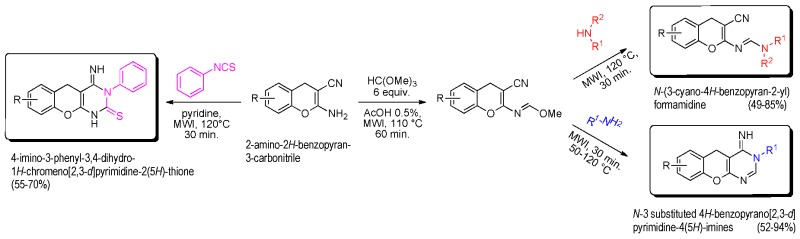


**Acknowledgments:** One of us (A.B.) wishes to thank the “Ministère de l’Enseignement Supérieur et de la Recherche de Tunisie” for the grant. Financial support of this program carried out under the French National Cancer Institute “Cancéropôle Grand Ouest” by “Valorisation des produits de la mer en cancérologie” contract, is gratefully acknowledged.

### 2.23. N-Acyclic Carbenes Synthesis, Characterization and their Applications as Anticancer Agents

LavrijsenMelanie AliagaVillacampaM. Dolores*GimenoM. Concepción*Departamento Química Inorgánica, Instituto de Síntesis Química y Catálisis Homogénea (ISQCH). Universidad de Zaragoza-CSIC, 50009 Zaragoza, España*Correspondence: dvilla@unizar.es (M.D.V.); gimeno@unizar (M.C.G.)

Gold drugs are well known and have been widely studied for their potential chemotherapeutic properties in anticancer treatments, although they have some limitations. (Bertrand, B., et al. *Dalton Trans.* **2014**, *43*, 4209–4219; Bertrand, B., et al. *J. Biol. Inorg. Chem.* **2015**, *20*, 1005–1020). Gold *N*-heterocyclic carbenes, especially NHC-Au(I) display high cytotoxicity in vitro (low micromolar to nanomolar) against a variety of human cancer cell lines with different degrees of selectivity. In the search for new alternatives, not only *N*-heterocyclic but N-acyclic carbenes must be explored. (Dada, O., et al. *J. Organomet. Chem.* **2017**, *840*, 30–37; Liu, W., et al. *Coordin. Chem. Rev.* **2016**, *329*, 191–213).

*N*-acyclic carbenes are easily accessible via the reaction between isocyanide gold compounds and different amines. The reaction between one of those derivatives with different thiol groups, in presence of K_2_CO_3_ as a deprotonating agent, has led to a family of gold(I) NAC thioderivatives with high cytotoxicity.

Biological activity of the different synthetized compounds was measured by MTT assays for different human cancer cell lines: A-549 (lung cancer), MiaPaca2 (pancreatic cancer), calculating their IC_50_ values, which were found in many cases to be less than six, being these results very promising.


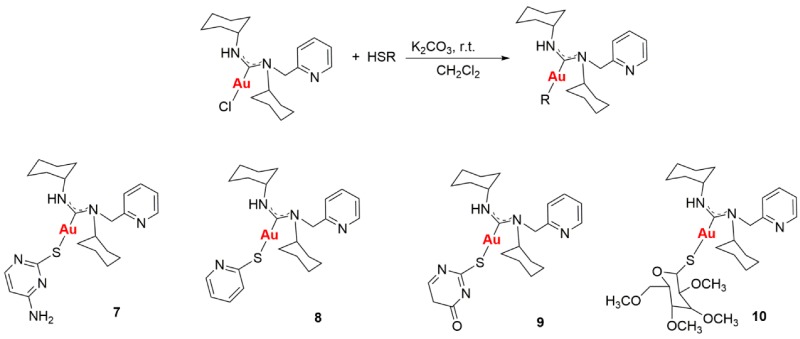

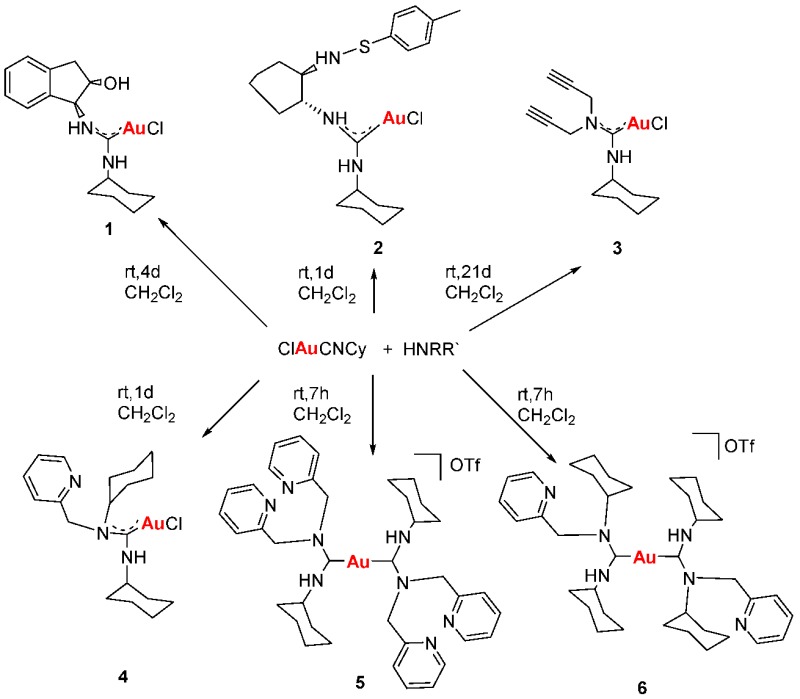


### 2.24. New Thiourea-Thiazolidine Complexes and Study of their Biological Activity

Salvador-GilDanielGimenoM. Concepción*Departamento de Química Inorgánica, Instituto de Síntesis Química y Catálisis Homogénea (ISQCH), Universidad de Zaragoza-CSIC, E-50009 Zaragoza, Spain*Correspondence: gimeno@unizar.es

Urea and thiourea scaffolds have been successfully used in drug design in recent years (Yao, J., et al. *Bioorg. Med. Chem.* **2012**, *20*, 2923–2929). Interestingly, other thiourea derivatives such as thiazolidines have attracted great attention more recently because their biological activity (Liu, Y., et al. *Bioorg. Med. Chem.* **2011**, *19*, 2342–2348).

It is known that the coordination with different metals such as gold or silver could induce better biological activity (Rackham, O., et al. *Biochem. Pharmacol.* **2007**, *74*, 992–1002). Therefore, we decided to coordinate these metal centers to the thioureas and to the heterocyclic compounds synthesized by us.

Although the formation of thiazolidines by reaction of propargylamine with isothiocyanates under harsh conditions has been previously reported, the reaction between propargylamines and isothiocyanates (Scheme 1), when the stoichiometry was 2:1, is new and it showed the formation of pioneering thiourea-thiazolidine derivatives. These new molecules are able to coordinate metal centers through its sulfur and nitrogen atoms, improving their biological activity. On the other hand, these complexes could work recognizing different target cells leading to higher selectivity.


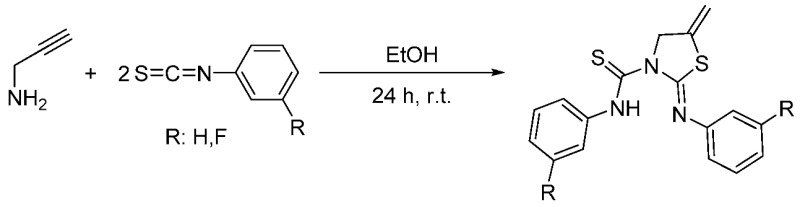


Finally, these compounds were tested with HeLa cells through the MTT assay. The results were not as successful as expected and only silver compounds showed good cytotoxic values as anticancer drugs. In contrast, gold compounds showed lower IC_50_ values than their respective organic ligands. Further studies will be performed to improve these activities and in order to develop better candidates for the treatment of cancer.

**Acknowledgments:** Authors thank the Ministerio de Economía y Competitividad (MINECO/FEDER CTQ2016-75816-C2-1-P), and DGA-FSE (E77) for financial support of this research.

### 2.25. Organocatalytic Synthesis of Chiral 1,4-Dihydropyridines with Potential Biological Properties

Auria-LunaFernando^1^Marqués-LópezEugenia^1^*GimenoM. Concepción^2^HerreraRaquel P.^1^*1Laboratorio de Organocatálisis Asimétrica, Departamento de Química Orgánica, Instituto de Síntesis Química y Catálisis Homogénea (ISQCH), CSIC-Universidad de Zaragoza, C/Pedro Cerbuna 12, 50009 Zaragoza, Spain2Departamento de Química Inorgánica, Instituto de Síntesis Química y Catálisis Homogénea (ISQCH), CSIC-Universidad de Zaragoza, C/Pedro Cerbuna 12, 50009 Zaragoza, Spain*Correspondence: mmaamarq@unizar.es (E.M.-L.); raquelph@unizar.es (R.P.H.)

The 1,4-dihydropyridine core is a widely studied privileged scaffold. Molecules containing this structure are well-known calcium channel blockers and are being used already as drugs in the treatment of heart diseases. Moreover, recent advances have demonstrated their potential to act against many other diseases. The recent research concerning their activity as multidrug-resistance reversing agents should be highlighted (Edraki, N., et al. *Drug Discov. Today* **2009**, *14*, 1058–1066). In the chemistry field, they are soft reducing agents and their chiral analogues have been used in asymmetric reductions with good results (Herrera, R. P. *Top. Curr. Chem.* **2016**, *374*, 29).

As shown before, these molecules contain a chiral center in their C4 position. The control of the selectivity in chemical transformations has been a crucial challenge in organic chemistry. Nowadays, is well known that the living matter can actually discern between stereoisomers of the same compound. Nevertheless, there are scarce examples of procedures leading to enantiomerically enriched 1,4-DHPs, being most of them based on the use of chiral auxiliaries or chiral resolutions (Auria-Luna, F., et al. *Adv. Synth. Catal.* **2017**, *359*, 2161–2175). Finding new and more environmental-friendly processes is also an interesting matter in chemistry, and organocatalytic procedures are a perfect tool to achieve this goal.

Herein, we report our recent advances in the development of new organocatalytic methodologies to produce enantiomerically enriched 1,4-DHPs (Auria-Luna, F., et al. *J. Org. Chem.* **2017**, *82*, 5516–5523). Interestingly, one of them, brings out another privileged scaffold, such as the oxindole motif (Auria-Luna, F., et al. *Molecules* **2015**, *20*, 15807–15826). Our methodologies could be perfect keystones leading to further research on the biological properties of these promising compounds.


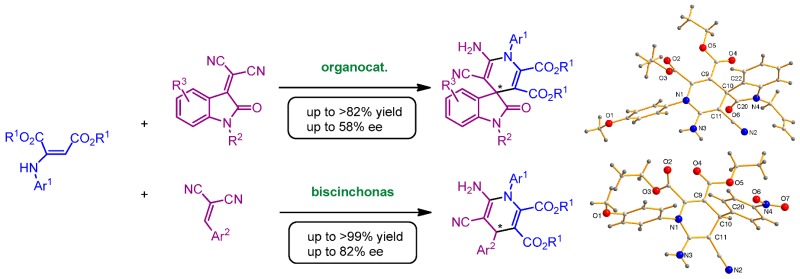


**Acknowledgments:** Authors thank the Ministerio de Economía, Industria y Competitividad (MINECO/FEDER CTQ2016-75816-C2-1-P and CTQ2017-88091-P), DGA-FSE (E77 and E104) and UZ (JIUZ-2017-CIE-05) for financial support of this research.

### 2.26. Synthesis of Luminescent Squaramide Monoesters: Cytotoxicity and Cell Imaging Studies in HeLa Cells

Fernández-MoreiraVanesa^1^Alegre-RequenaJuan V.^2^HerreraRaquel P.^2^*MarzoIsabel^3^GimenoM. Concepción^1^*1Departamento de Química Inorgánica, Instituto de Síntesis Química y Catálisis Homogénea (ISQCH), Universidad de Zaragoza-CSIC, E-50009 Zaragoza, Spain2Laboratorio de Organocatálisis Asimétrica, Departamento de Química Orgánica, Instituto de Síntesis Química y Catálisis Homogénea (ISQCH), Universidad de Zaragoza-CSIC, E-50009 Zaragoza, Spain3Departamento de Bioquímica y Biología Molecular y Celular, Universidad de Zaragoza, E-50009 Zaragoza, Spain*Correspondence: raquelph@unizar.es (R.P.H.); gimeno@unizar.es (M.C.G.)

The squaramide motif has gained increased interest in medicinal chemistry, being considered a valuable scaffold for drug design (Quintana, M., et al. *Med. Chem. Commun.* **2016**, *7*, 550–561). In contrast, the study of squaramide monoesters as cytotoxic agents or for cell imaging has been overlooked in the literature so far, and no examples of potential anticancer agents have been previously reported.

Novel luminescent squaramide monoesters functionalized with different fluorophore groups have been synthesized and explored in cell imaging for the first time (Alegre-Requena, J., et al. *RSC Adv.* **2015**, *5*, 33450–33462). In view of the excellent emission properties of all tested compounds, a series of experiments were undertaken to test their cytotoxic activity and viability as specific cell imaging agents in *human HeLa cervical cancer cells*. Cytotoxicity studies performed revealed high activity for some of these novel structures, highlighting the importance of the fluorescent fragment in the efficiency of these promising anticancer agents (Fernández-Moreira, V., et al. *RSC Adv.* **2016**, *6*, 14171–14177).


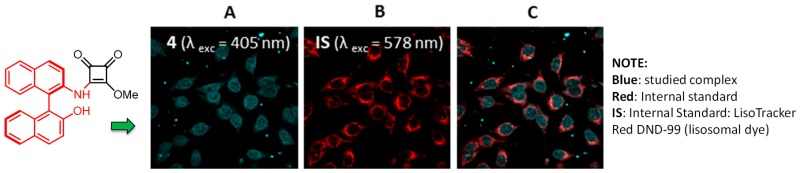


Fluorescence cell microscopy pointed out the different biodistribution behavior depending on the fluorescent moiety, and both lysosomal and nuclear localization has been observed, which highlights and proposes the possibility for chiral and nonplanar bioprobes, as in the case of compound depicted in the figure, to exert a chiral recognition within the nucleus. Since the squaramide functionality provides a way to increase the transport ability of a receptor without significantly increasing the lipophilicity, it offers an ideal platform for designing future anion transporters. Further related studies with this research are ongoing in our lab and will be published in due course.

**Acknowledgments:** Authors thank the Ministerio de Economía y Competitividad (MINECO/FEDER CTQ2013-48635-C2-1-P, SAF2013-48626-C2-2-R, CTQ2016-75816-C2-1-P and CTQ2017-88091-P), and DGA-FSE (E77 and E104) for financial support of this research.

### 2.27. Optimization of Conditions for the Chromatographic Isolation of Isohexenyl Naphthazarin Derivatives from the Rhizome Callus of Echium Vulgare

StanićPetar*VukovićNenadFaculty of Science, University of Kragujevac, Radoja Domanovića 12, 34000 Kragujevac, Serbia*Correspondence: rope034@gmail.com

Recently, naphthazarin derivatives have attracted huge attention due to their broad variety of biological activities, which include wound healing, anti-inflammatory effects, antitumor, anti-microbial and antithrombotic activity, etc. These compounds are not only important for their biological activity (and medicinal applications), but also in industry (food colorings, cosmetics, wood coatings, etc.). In this study, three naphthazarin derivatives (deoxyshikonin, acetylshikonin and β-hydroxylisovalerylshikonin) have been identified after isolation from the plant *Echium vulgare*, which is abundant in the Serbian territory as a wild and cultivated species. The plant itself has been characterized as having a low naphthazarin pigment content compared to *Onosma visianii*, *Onosma paniculata*, *Alkanna tinctoria* and *Lithospermum erythrorhizon*, which are used for mass production of these pigments. The isolation method described in this study makes possible preparation of a concentrated *Echium vulgare* extract. This concentrated extract can complement the plant extracts with higher naphthazarin pigment content that either are non-native or endangered in Europe.


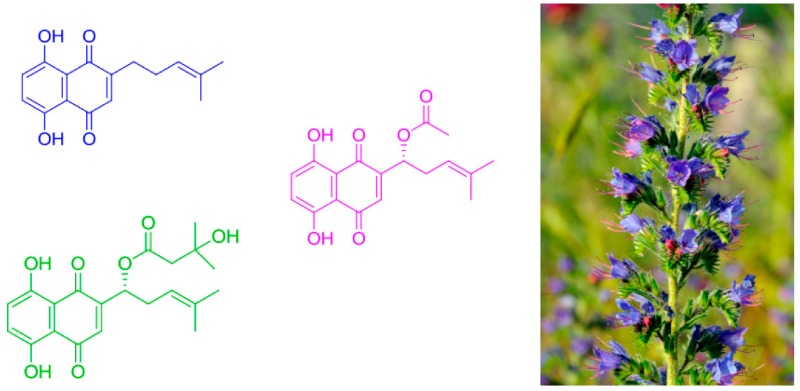


### 2.28. Synthesis of Squaramides with Anti-tumor Activity

Alegre-RequenaJuan V.^1^Marqués-LópezEugenia^1^HerreraRaquel P.^1^*QuintanaMireia^2^TriolaGemma^2^*1Laboratorio de Organocatálisis Asimétrica, Departamento de Química Orgánica, Instituto de Síntesis Química y Catálisis Homogénea (ISQCH), CSIC-Universidad de Zaragoza, E-50009 Zaragoza, Spain2Biomedicinal Chemistry Department, Institute of Advanced Chemistry of Catalonia (IQAC), CSIC, E-08034 Barcelona, Spain*Correspondence: raquelph@unizar.es (R.P.H.); gemma.triola@iqac.csic.es (G.T.)

In this study, the cytotoxic effects of different squaramides were tested against diverse cancer cells, such as HGC-27, HeLa, T98 and U87 cells, and non-cancer cells, such as EK293, MDCK and Vero cells. We found a disubstituted squaramide that showed an IC_50_ of 1.8 μM against HGC-27 cells, which is considerably lower than the IC_50_ observed in the rest of the cell lines (Quintana, M., et al. *MedChemComm* **2016**, *7*, 550–561).


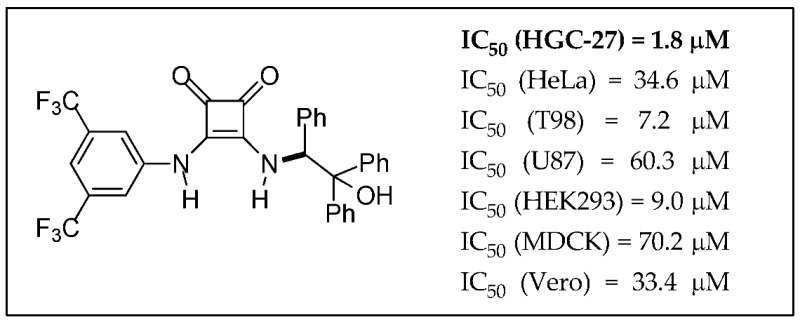


Furthermore, the mechanism of action of this compound was evaluated. The results indicate that the decrease in cell viability produced by the squaramide is probably caused by G_0_/G_1_ cell cycle arrest and caspase-mediated apoptosis. Additionally, the cell death produced by this compound is accompanied by autophagy induction having a protective effect and no signs of cathepsin-mediated cell death or necroptosis have been observed. The creation of compounds that trigger a specific cell death subroutine is preferred since it might avoid potential side-effects and nonspecific cytotoxic effects. Therefore, this squaramide and its derivatives could be promising molecules for the treatment of gastric carcinoma.

**Acknowledgments:** Funding from the Marie Curie Career Integration Grants (Grant PCIG12-GA-2012-333835), the Max Planck Society (Partner Groups), the Ministerio de Economia y Competitividad (Grant CTQ2013-44334-P and CTQ2017-88091-P), the University of Zaragoza (JIUZ-2014-CIE-07 and JIUZ-2017-CIE-05), the High Council of Scientific Investigation (CSIC) (PIE-201580I010) and Government of Aragon DGA (Research Group E-104) is gratefully acknowledged.

### 2.29. Arylidene Ketones with Potent Trypanosomicidal Activity that Causes Late Apoptosis/Necrosis Like Nifurtimox

AguileraElena^1^*MosquilloFlorencia^2^PérezLeticia^2^CerecettoHugo^1^,^3^ÁlvarezGuzmán^4^GonzálezMercedes^1^1Grupo de Química Medicinal, Facultad de Ciencias Universidad de la República, Iguá. 4225, 11400 Montevideo, Uruguay2Laboratorio de Interacciones Moleculares, Facultad de Ciencias, Universidad de la República, Iguá, 4225, 11400 Montevideo, Uruguay3Área de Radiofarmacia, Centro de Investigaciones Nucleares, Universidad de la República, Mataojo S/N, 11400 Montevideo, Uruguay4Laboratorio de Moléculas Bioactivas, Centro Universitario Regional Litoral Norte, Universidad de la República, Rute 3 km 363, 60000 Paysandú, Uruguay*Correspondence: eaguilera@fcien.edu.uy

Chagas disease is caused by the parasite *Trypanosoma cruzi (T. cruzi)* and it remains the major parasitic disease in Latin America. The chemotherapy employed to control the parasitic infection is based on two drugs: nifurtimox (**Nfx**) and benznidazole (**Bnz**), requiring long-term treatment that can give rise to severe side effects. They are not active against all *T. cruzi* strains, exhibit low efficiency in long-term chronic infections, and are mutagenic. The search of new drugs is an urgent need (Cabrera M., et al. *Toxicol. Lett.* **2009**, *190*, 140).

In this work, we used three symmetrical diarylideneketones **1**–**3** containing thiophene and furan moieties. These molecules showed good to excellent trypanosomicidal activity and selectivity to the parasite, affected cruzipain, a proteolytic enzyme of the parasite, and the glycolytic enzyme, triosephosphate isomerase of *T. cruzi* (TcTIM) without affecting human´s TIM and showing effectiveness in protecting infected mice without toxic effects in vivo. Arylidene ketones **1** and **2** cause, after 24 h, late apoptosis/necrosis at a concentration of 20 times the value of its IC_50_ (approximately 80% of late apoptosis/necrosis) like **Nfx**. What happens with compound **3** should be further studied since no death by apoptosis or necrosis is observed at a dose of 20 times the value of their IC_50_ like **Bnz** (Aguilera, E., et al. *ChemMedChem* **2016**, *11*, 1328–1338). These results were obtained by flow cytometry and ^1^H-NMR (Vermes, I., et al. *J. Immunol. Methods* **1995**, *184*, 39–51; Benitez, D., et al. *Parasitology* **2012**, *139*, 506–515).


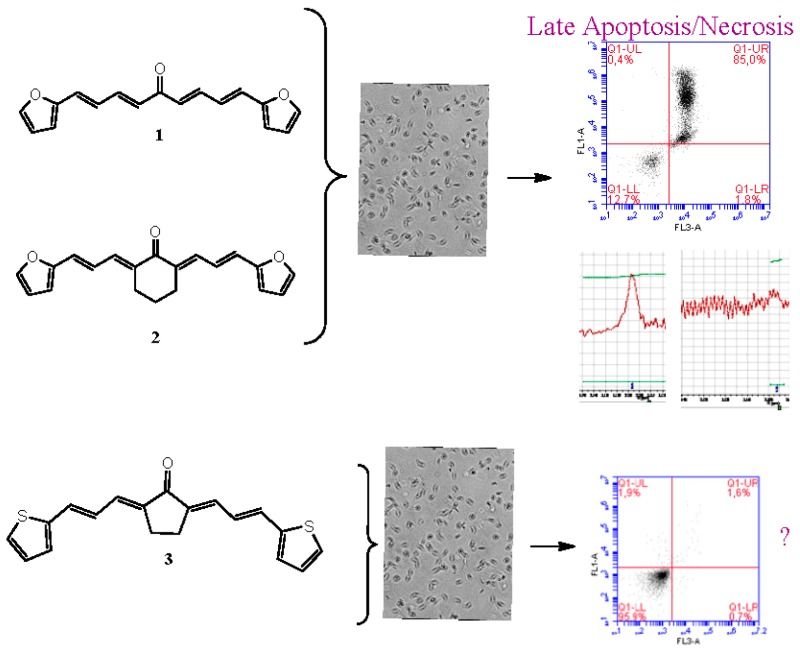


**Acknowledgments:** This work was funded by Comision Sectorial de Investigacion Cientifica (CSIC)-Universidad de la Republica (CSIC No 661) and Programa de Desarollo de las Ciencias Básicas (PEDECIBA). Elena Aguilera has scholarship from Agencia Nacional de Investigación e Innovación (POS_NAC_2016_1_129945).

### 2.30. Identification of a Novel Potent and Selective Anti-Trichomonas Vaginalis Agent among Libraries of Bisbenzimidazoles

EyndeJean Jacques Vanden^1^*MayenceAnnie^1^HuangTien L.^1^YarlettNigel^2^,^3^1Division of Basic Pharmaceutical Sciences, College of Pharmacy, Xavier University of Louisiana, New Orleans, LA 70125, USA2Department of Chemistry and Physical Sciences, Pace University, New York, NY 10038, USA3Haskins Laboratories, Pace University, New York, NY 10038, USA*Correspondence: jean-jacques.vandeneynde@ex.umons.ac.be

Small libraries of bisbenzimidazoles structurally related to pentamidine have been synthesized and evaluated against different species of parasites. 2,2′-[1,3-Propanediylbis(oxy-1,3-phenylene)]bis-1*H*-benzimidazole emerged as a potent and selective anti-*Trichomonas vaginalis* agent from in vitro and in vivo studies. In particular, in vitro under aerobic conditions the compound was more active than metronidazole against both metronidazole-susceptible (C1) and -refractory (085) isolates of *Trichomonas vaginalis*. In vivo, it cured a subcutaneous mouse model infection using both kinds of isolates.


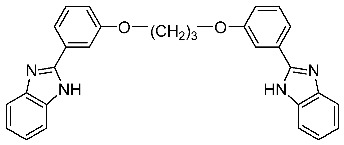


## 3. Posters

### 3.1. Novel Racemic and Enantiopure Amino-Fluorene-Methanol Coumponds with Antimalarial Activities

SchneiderJérémyDassonville-KlimptAlexandraDemailly-MulliéCatherineSonnetPascal*Laboratoire de Glycochimie des Antimicrobiens et des Agroressouces (LG2A), UMR CNRS 7378, Université de Picardie Jules Verne, UFR de pharmacie, 1 rue des Louvels, F-80037 Amiens CEDEX 01, France*Correspondence: pascal.sonnet@u-picardie.fr

Malaria is a neglected tropical disease that remains a leading cause of morbidity and mortality among the world’s poorest populations. More than 100 tropical and sub-tropical countries are endemic for this infectious disease. Pregnant women and children are the most sensitive to this infection and, in 2015, 429,000 people died. Among the five species of *Plasmodium* responsible for human malaria, *P. falciparum* is the parasite which causes the most serious form of the disease. More recent efforts focused on the development of antimalarial vaccines and since 2001, World Health Organization (WHO) recommends artemisinin-based combination therapies (ACTs). In drugs resistance areas, several antimalarial drugs, such as aminoalcohol-aryl (mefloquine (MQ), lumefantrine (LM)), are currently used in combination with artemisinin derivatives. However, the emergence of multi-drug-resistant parasites decreases efficacy of ACTs. Thus, the design of new active compounds on *Plasmodium*-resistant strains is urgent.

We have previously developed an asymmetric synthesis to prepare 4-aminoalcohol-quinoline enantiomers (AQ) as MQ analogs. They were active on nanomolar range against 3D7 (chloroquine-sensitive) and W2 (chloroquine-resistant) *P. falciparum* strains. Interestingly, (*S*)-enantiomers displayed 2 to 15-fold increased activity as compared to their (*R*)-counterparts. During the *Plasmodium* intra-erythrocytic asexual stages, hemozoin formation and the oxidative and glutathione-dependent degradation of heme are inhibited by these aminoalcohol-aryls (MQ, LM). Currently, their mechanisms of actions are not totally clear and remain to be explored. In continuation of our work, we are interested to study the change of heterocycle (fluorene vs quinoline) on the antimalarial activity. We focus on the design and the preparation of novel racemic and enantiopure aminoalcohol-fluorene derivatives (AFM) as LM analogs. The evaluation of their antiplasmodial activity against *P. falciparum* and their corresponding cytotoxicity is under progress.

**Acknowledgments:** J.S. was the recipient of a grant from DGA (Direction Générale de l’Armement, Ministère de la Défense, France) and Région Picardie.

### 3.2. Asymetric Synthesis of 3,6-Disubstituted Dioxopiperazines with Potential Siderophore Properties

GarnerinTimothéeDassonville-KlimptAlexandra*SonnetPascal*Laboratoire de Glycochimie des Antimicrobiens et des Agroressouces (LG2A), UMR CNRS 7378, Université de Picardie Jules Verne, UFR de pharmacie, 1 rue des Louvels, F-80037 Amiens CEDEX 01, France*Correspondence: alexandra.dassonville@u-picardie.fr (A.D.-K.); pascal.sonnet@u-picardie.fr (P.S.)

Antibiotic resistance is an emerging disease and a real problem of health. Resistance of Gram negative bacteria such as *Acinetobacter baumannii* and *Escherichia coli* to conventional antibiotics leads to therapeutic failure and requires new antibiotherapies. The use of the iron transport systems is one of the most promising strategies to overcome this resistance phenomenon. These specific routes of entry, essential for the survival of the microorganisms, allow ferric siderophore complexes to carry iron within the bacteria.

These systems allow the introduction of antibacterial agents (conjugates antibiotic-siderophore) or toxic complexes (gallium complexes) into the bacteria to kill them. Rhodotorulic acid (RA) is a siderophore transported by TonBox dependant Fhu receptors. These kinds of receptors are expressed by *Acinetobacter baumannii* and *Escherichia coli*. RA is dioxopiperazine iron chélater with hydroxamate as iron ligands and two asymmetric centers (*S*,*S*-configuration). This spatial orientation is essential for the Fhu receptors recognition. We have previously reported the asymmetric synthesis of 3-substituted 2-oxopiperazines. Herein, we present an original and a convergent strategy to synthesize RA and corresponding 3,6-disubstituted analogues. Siderophore-like test and measurement of the complexing strength of these compounds will be carried out.


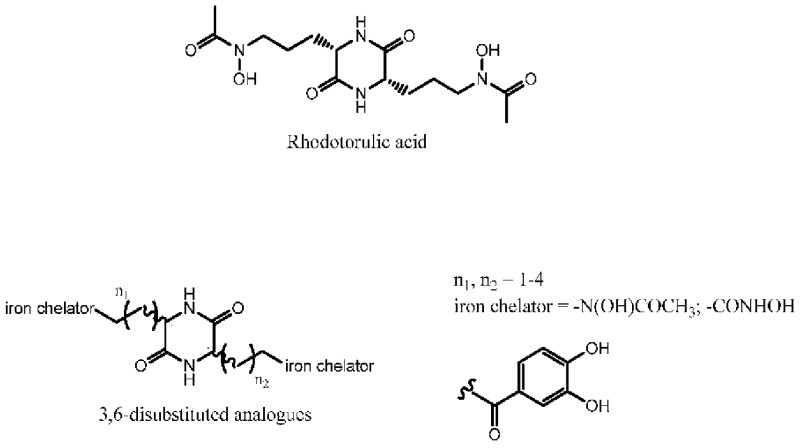


**Acknowledgments:** T.G. was the recipient of a grant from Région Picardie.

### 3.3. Design of New Polymyxins with Reduced Nephrotoxicity

SegoviaRoser^1^SoléJudith^1^ManresaAngeles^2^CajalYolanda^3^,^4^RabanalFrancesc^1^*1Section of Organic Chemistry, Dept of Inorganic and Organic Chemistry, Faculty of Chemistry, University of Barcelona, 8007 Barcelona, Spain2Laboratory of Microbiology, Faculty of Pharmacy, University of Barcelona, 8007 Barcelona, Spain3Department of Physical Chemistry, Faculty of Pharmacy, University of Barcelona, 8007 Barcelona, Spain4Institute of Nanoscience and Nanotechnology (IN2UB), University of Barcelona, 8007 Barcelona, Spain*Correspondence: frabanal@ub.edu

There is a clear unmet medical need in the field of infectious diseases. A major goal to fight resistant bacteria involves the design, discovery and development of new antibiotics particularly against multi-drug-resistant strains. Polymyxins, an old class of antimicrobial cyclic lipopeptides highly potent against therapeutically relevant Gram-negative bacteria, are now used as last resort antibiotics in hospitals because of their nephrotoxicity and neurotoxicity that require careful monitoring of the patient (Rabanal, F., et al. *Nat. Prod. Rep.* **2017**, *34*, 886–908). Our group has embarked in a project to design and develop new polymyxins devoid of toxicity problems using a versatile and chemically accessible scaffold structure (Rabanal, F., et al. *Sci. Rep.* **2015**, *5*, 10558). Compounds show a remarkable activity against Gram-negative bacteria. Herein, the last results of our recently designed polymyxin analogs are presented.


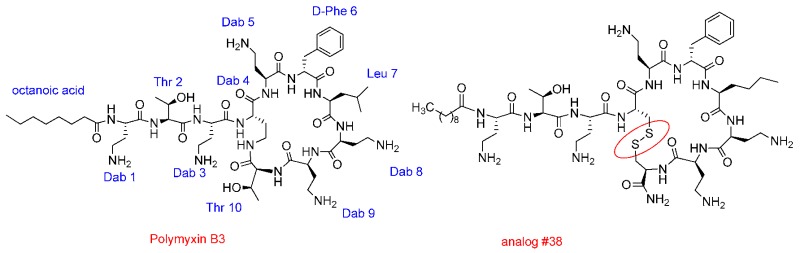


**Acknowledgments:** This research was supported by the University of Barcelona, Fundació Bosch i Gimpera, Xarxa de Referència en Biotecnologia, Generalitat de Catalunya (grant 2016LLAV00018, FEDER funds) and the European Institute of Innovation and Technology (EITHealth). FR, AM and YC are members of the ENABLE (European Gram-Negative Antibacterial Engine) consortium.

### 3.4. QSAR Model: Prediction of the Clastogenic Potential of 3-Arylcoumarins

YordiEstela Guardado^1^,^2^PérezAmaury^3^LeónLianne^2^MolinaEnrique^1^,^2^SantanaLourdes^1^UriarteEugenio^1^,^4^MatosMaria João^1^*1Universidad de Santiago de Compostela, Facultad de Farmacia, Campus vida, 15782 Santiago de Compostela, Spain2Universidad de Camagüey Ignacio Agramonte Loynaz, Facultad de Ciencias Aplicadas, Circunvalación Norte Km 5 1/2, 74650 Camagüey, Cuba3Universidad Estatal Amazónica, Facultad de Ciencias de la Tierra, Km 2 1/2 vía Puyo a Tena (Paso Lateral), Puyo, Ecuador4Instituto de Química Aplicada, Universidad Autónoma de Chile, Pedro de Valdivia 425, 7500912 Santiago, Chile*Correspondence: mariajoao.correiapinto@usc.es

Drug discovery is a challenging task for researchers, due to the complexity of biomolecules involved in pathologic processes. Design and development of efficient drugs is still urgent for several diseases. Cheminformatics tools are useful to better understand the interaction between new chemical entities and their targets. We studied a selected series of 3-arylcoumarins with antioxidant potential, and how their chemical features can contribute for the clastogenic activity. A virtual screening, based on the TOPSMODE approach, using a clastogenic model, was performed. The results suggest that the presence and position of hydroxyl groups in the scaffold is important for the activity. This communication is focused on cheminformatics, and its applications in drug effectiveness and safety.

**Acknowledgments:** The authors thank the partial financial support of the University of Santiago de Compostela and the University of Camagüey Ignacio Agramonte Loynaz. MJM thanks Galician Plan of research, innovation and growth 2011–2015 (Plan I2C, ED481B 2014/086-0).

### 3.5. Effect of Interaction with Micellar Media on Spectral Properties of Some Amphiphilic Porphyrins

BoscencuRica^1^SocoteanuRadu^2^*NaceaVeronica^1^FerreiraLuís Filipe Vieira^3^1Faculty of Pharmacy, “Carol Davila” University of Medicine and Pharmacy, 6 Traian Vuia St., 020956 Bucharest, Romania2“Ilie Murgulescu” Institute of Physical Chemistry, Romanian Academy, 202 Splaiul Independenţei, Bucharest 060021, Romania3Centro de Química-Física Molecular, Institute of Nanosciences and Nanotechnology, Instituto Superior Técnico Av. Rovisco Pais, 1049-001 Lisboa, Portugal*Correspondence: psradu@yahoo.com

Porpyrins—compounds used in Photodynamic Therapy—easily form aggregates, which have a lower ability for localization at cellular level and consequently decrease the therapeutic effect. So, before pharmaceutical formulation, it is necessary to evaluate the spectral and aggregation properties of these compounds in membrane mimetic media, in order to determine the factors that modulate porphyrin−membrane interactions. The present study includes spectral evaluation of 5-(2-hydroxyphenyl)-10,15,20–*tris*-(4-carboxymethylphenyl) porphyrin and its complexes with Zn(II) and Cu(II) in TX-100/water and TX-100/cyclohexane micelles.


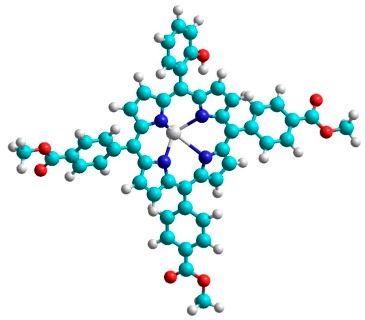


Regarding to spectral behavior of the studied porphyrins, the experimental results confirms the fact that incorporation in micelles will facilitate a better delivery to the cellular target without determine significant change in their photophysical profile.

**Acknowledgments:** This study was supported by ERA.NET projects: BIOMARK (ctr. 7030/2010) and NANOTHER (ctr. 53/2016 and 54/2016) of the Romanian Ministry of Education and Research.

### 3.6. Microtubule-Destabilising Actions of Piperlongumine and Analogues

O’BoyleNiamh M.^1^*ZistererDaniela M.^2^MeeganMary J.^1^1School of Pharmacy & Pharmaceutical Sciences, Trinity College Dublin, Dublin 2, Ireland2School of Biochemistry & Immunology, Trinity College Dublin, Dublin 2, Ireland*Correspondence: niamh.oboyle@tcd.ie

Piperlongumine is a natural amide alkaloid isolated from the plant species *Piper longum* L. It is known to be cytotoxic (Bezerra, D.P. et al. *Toxicol. Vitr.* **2007**, *21*, 1–8) and has been reported to selectively kill cancer cells by targeting the stress response to reactive oxygen species (ROS) (Raj, L., et al. *Nature* **2011**, *475*, 231–234). The use of small molecules to target cancer by altering cell levels of ROS is emerging area of research and there is huge potential for further exploration in this area (Schumacker, P.T. *Cancer Cell* **2006**, *10*, 175–176).

Piperlongumine is structurally similar to a number of microtubule-destabilizing agents, including combretastatin A-4 and related chalcones. This project focuses on the effects of piperlongumine on tubulin, a protein that is the main constituent of microtubules. The synthesis of analogues of piperlongumine was carried out to establish a structure-activity relationship for microtubule-destabilizing activity. The novel compounds were screened for their antiproliferative effects in breast cancer cells and normal breast cells. The effect of the piperlongumine analogues on ROS levels was also investigated.


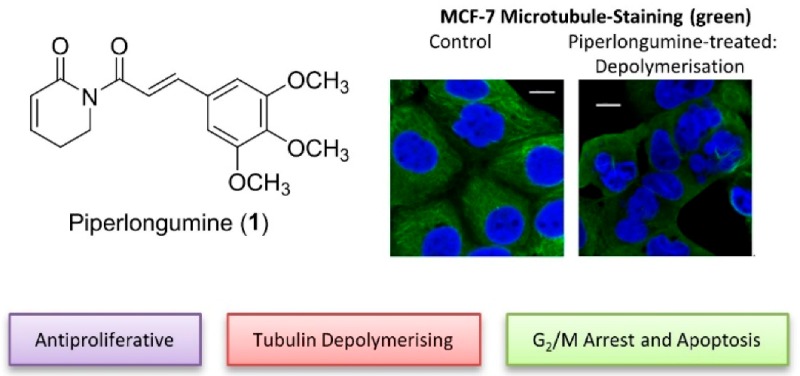


**Acknowledgments:** This work was supported by the Irish Research Council (GOIPD/2013/188; NMO’B). The Trinity Biomedical Sciences Institute is supported by a capital infrastructure investment from Cycle 5 of the Irish Higher Education Authority’s Programme for Research in Third Level Institutions (PRTLI). The skilful technical assistance of Gavin McManus (confocal microscopy) and Barry Moran (flow cytometry) are gratefully acknowledged.

### 3.7. Lead Optimization of Benzoxepin-Type Selective Estrogen Receptor (ER) Modulators and Downregulators with Subtype-Specific ERα and ERβ Activity

O’BoyleNiamh M.^1^*ZistererDaniela M.^2^MeeganMary J.^1^1School of Pharmacy & Pharmaceutical Sciences, Trinity College Dublin, Dublin 2, Ireland2School of Biochemistry & Immunology, Trinity College Dublin, Dublin 2, Ireland*Correspondence: niamh.oboyle@tcd.ie

Estrogen receptor-α (ERα) is an important target for the design of drugs such as tamoxifen and fulvestrant. Three series of ER-ligands based on the benzoxepin scaffold structure were synthesized—series I containing an acrylic acid, series II with an acrylamide and series III with a saturated carboxylic acid substituent. These compounds were shown to be high affinity ligands for the ER with nanomolar IC_50_ binding values. Series I acrylic acid ligands were generally ERα selective. In particular, a compound (**1**) featuring a phenylpenta-2,4-dienoic acid substituent was shown to be antiproliferative and downregulated ERα and ERβ expression in MCF-7 breast cancer cells. Interestingly, from series III, a phenoxybutyric acid derivative compound (**2**, below) was not antiproliferative and selectively downregulated ERβ. A docking study of the benzoxepin ligands was undertaken. Compound **1** is a promising lead for development as a clinically relevant SERD, whilst compound **2** will be a useful experimental probe for helping to elucidate the role of ERβ in cancer cells.


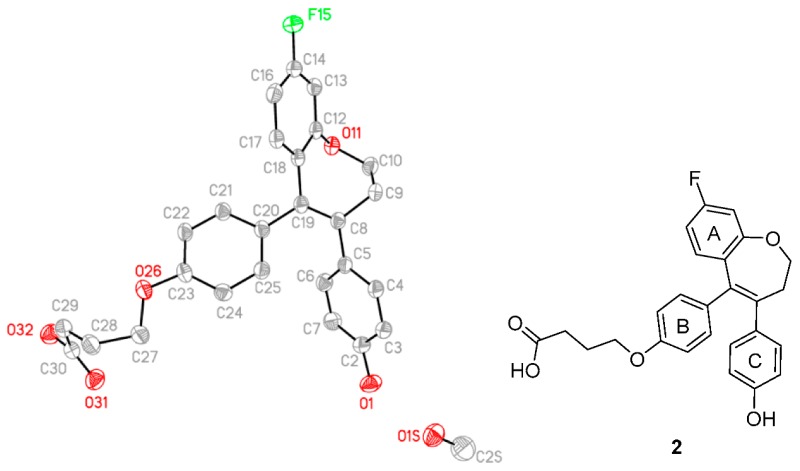


**Acknowledgments:** We are very grateful to Professor Richard Hochberg (Yale University Medical School) for the generous gift of the Ishikawa cells and to Susan McDonnell (UCD School of Biomedical Engineering) for the kind gift of MCF10a cells. This work was supported through funding from the Trinity College IITAC research initiative (HEA PRTLI), Enterprise Ireland (EI) and the Irish Research Council (GOIPD/2013/188; NMO′B). The Trinity Biomedical Sciences Institute is supported by a capital infrastructure investment from Cycle 5 of the Irish Higher Education Authority’s Programme for Research in Third Level Institutions (PRTLI).

### 3.8. Novel N-(2-Mercaptobenzenesulfonyl)guanidine Derivatives Modified by Nitrogen-Containing Heterocycles—Synthesis and Antiproliferative Activity Against Human Cancer Cell Lines

PogorzelskaAneta^1^*SławińskiJarosław^1^KawiakAnna^2^,^3^JasińskaJoanna^1^1Department of Organic Chemistry, Medical University of Gdansk, Faculty of Pharmacy with Subfaculty of Laboratory Medicine, 80-416 Gdansk, Poland2Department of Biotechnology, Intercollegiate Faculty of Biotechnology UG-MUG, 80-307 Gdansk, Poland3Department of Human Physiology, Medical University of Gdansk, Faculty of Health Sciences, 80-210 Gdansk, Poland*Correspondence: anetapogorzelska@gumed.edu.pl

The *N*-(2-mercaptobenzenesulfonyl)guanidine derivatives **9**–**27** have been obtained by the reaction of the appropriate *N*-(2-mercaptobenzenesulfonyl)cyanamide potassium salt **1**–**8** with 1-aminopiperidine, 4-aminomorpholine or 1-amino-4-methylpiperazine.


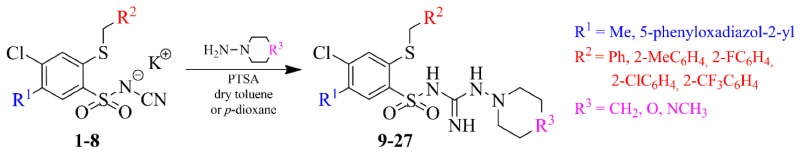


The compounds **9**–**27** have been evaluated in vitro for an activity against HCT-116 (colon carcinoma), MCF-7 (breast cancer) and HeLa (cervical cancer). The derivatives containing 4-methylpiperazine ring (R^3^ = NCH_3_) were the most potent growth inhibitors. The best activity achieved IC_50_ ≤ 15 μM for compounds with R^1^ = Me, R^2^ = 2-FC_6_H_4_. Moderate antiproliferative effect (IC_50_~40 μM) was observed for derivatives with piperidine residue (R^3^ = CH_2_). Compounds with morpholine fragment did not show significant growth inhibition (R^3^ = O).

### 3.9. Synthesis of Novel N-{[4-(1,2,3-Triazol)pyridin-3-yl]sulfonyl}urea Derivatives with Potential Anticancer Activity

SzafrańskiKrzysztof^1^*SławińskiJarosław^1^KawiakAnna^2^1Department of Organic Chemistry, Faculty of Pharmacy with Subfaculty of Laboratory Medicine, Medical University of Gdańsk, Al. Gen. J. Hallera 107, 80-416 Gdańsk, Poland2Department of Biotechnology, Intercollegiate Faculty of Biotechnology, University of Gdańsk and Medical University of Gdańsk, ul. Abrahama 58, 80-307 Gdańsk, Poland*Correspondence: k.szafranski@gumed.edu.pl

Diarylsulfonylureas (DSU) constitute a group of compounds which, unlike the *N*-alkyl sulfonylurea derivatives exhibit activity against a broad spectrum of syngeneic rodent solid tumors and human tumor xenografts. In our previous studies we have proved a significant potential of antitumor activity of 1-(4-substituted pyridine-3-sulfonyl)-3-phenylureas strongly affected by substituent at the position 4 of the pyridinesulfonamide scaffold (Szafrański K., et.al. *Molecules* **2015**, *20*, 12029–12044; Sławiński J, et al. *Eur. J. Med. Chem.* **2013**, *69*, 701–710).

Therefore, we have undertaken the synthesis (as presented on the scheme) of novel series of *N*-{[4-(1H-1,2,3-triazol-4-yl)methylamino/thio)pyridin-3-yl]sulfonyl}urea derivatives, and evaluation in MTT assay of their effects on the growth of human cancer cell lines: colon cancer HCT-116, breast cancer MCF-7 and cervical cancer HeLa.


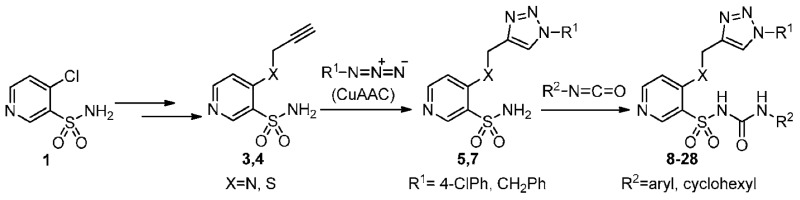


### 3.10. First Advances in the Asymmetric Synthesis of Biologically Active 2-Amino-3-cyano-4H-chromen-4-yl Phosphonates

SonsonaIsaac G.Muñiz-BustínSandraMarqués-LópezEugeniaHerreraRaquel P.*Laboratorio de Organocatálisis Asimétrica, Departamento de Química Orgánica, Instituto de Síntesis Química y Catálisis Homogénea (ISQCH) CSIC-Universidad de Zaragoza, C/ Pedro Cerbuna 12, 50009 Zaragoza, Spain*Correspondence: raquelph@unizar.es

The 2-amino-3-cyanochromene scaffold can be found in pharmacological agents like the antitumor crolibulin and the pro-apoptotic HA14-1. Recent studies have also shown that chromene phosphonyl derivatives exhibit different biological properties including antimicrobial, antifungal, antioxidant (Rajasekhar, M., et al. *Chem. Pharm. Bull.* **2012**, *60*, 854–858) and anticancer activity (Kalla, R.M.N., et al. *Eur. J. Med. Chem.* **2014**, *76*, 61–66). Since it is known that each enantiomer could present distinct biological response, the present research explores the use of chiral organocatalysts in the asymmetric synthesis of these interesting derivatives. To date, preliminary results obtained following two organocatalytic pathways, using diarylphosphonates (Pudovik reaction) and trialkylphosphites (Abramov reaction) as reagents, have shown significant chiral induction by the organocatalyst, achieving the first enantio-enriched mixtures of these compounds.


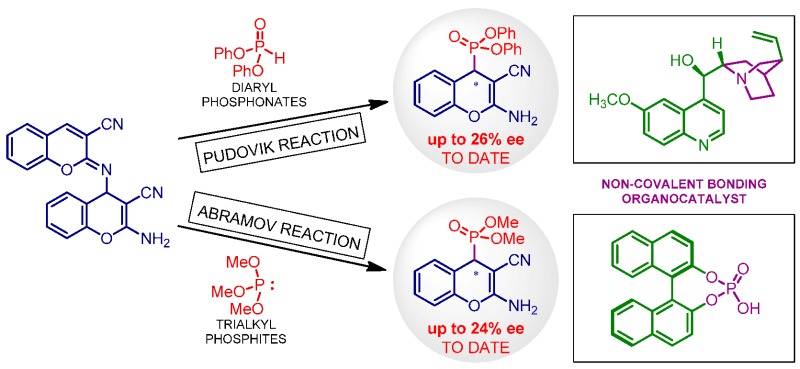


**Acknowledgments:** Authors thank the Ministerio de Economía, Industria y Competitividad (MINECO/FEDER CTQ2017-88091-P), DGA-FSE (E77 and E104) and UZ (JIUZ-2017-CIE-05) for financial support of this research.

## 4. Conclusions

The third International Electronic Conference on Medicinal Chemistry was a successful event having gathered around 300 authors from 34 different countries (Austria, Belgium, Brazil, Bulgaria, China, Cuba, Ecuador, Egypt, Finland, France, Germany, Greece, India, Indonesia, Ireland, Japan, Latvia, Macedonia, Mexico, Morocco, Nigeria, Pakistan, Poland, Portugal, Romania, Russia, Serbia, South Africa, Spain, Switzerland, Ukraine, United Kingdom, United States of America, and Uruguay). The virtual exhibition hall hosted 20 media partners.

The award for the best presentation, as selected by the Scientific Advisory Committee, was given to the group of Jalal Soubhye (Laboratoire de Chimie Pharmaceutique Organique, Faculté de Pharmacie, Université Libre de Bruxelles, Campusplaine, CP 205/5, 1050 Brussels, Belgium) for their work entitled: “Dual Anti-Inflammatory and Anti-Bacterial Effects of Phenylhydrazide and Phenylhydrazone Derivatives”.

Thanks to the support of the organizers, the sponsors, and the confidence of the participants, we are proud to announce that the fourth International Conference on Medicinal Chemistry will be held in November 2018 on www.sciforum.net/conference/ecmc-4. We hope that you will have the opportunity to attend, as authors or visitors.

## Supplementary Materials

The full presentations are available online at www.sciforum.net/conference/ecmc-3.
